# Genotyping-by-sequencing targets genic regions and improves resolution of genome-wide association studies in autotetraploid potato

**DOI:** 10.1007/s00122-024-04651-8

**Published:** 2024-07-09

**Authors:** Sanjeev Kumar Sharma, Karen McLean, Peter E. Hedley, Finlay Dale, Steve Daniels, Glenn J. Bryan

**Affiliations:** 1 Cell and Molecular Sciences, The James Hutton Institute, Invergowrie, Dundee, DD2 5DA UK; 2 Cygnet PB Ltd, Cambridge, CB21 6AS UK

## Abstract

**Key message:**

**De novo genotyping in potato using methylation-sensitive GBS discovers SNPs largely confined to genic or gene-associated regions and displays enhanced effectiveness in estimating LD decay rates, population structure and detecting GWAS associations over ‘fixed’ SNP genotyping platform. Study also reports the genetic architectures including robust sequence-tagged marker–trait associations for sixteen important potato traits potentially carrying higher transferability across a wider range of germplasm.**

**Abstract:**

This study deploys recent advancements in polyploid analytical approaches to perform complex trait analyses in cultivated tetraploid potato. The study employs a ‘fixed’ SNP Infinium array platform and a ‘flexible and open’ genome complexity reduction-based sequencing method (GBS, genotyping-by-sequencing) to perform genome-wide association studies (GWAS) for several key potato traits including the assessment of population structure and linkage disequilibrium (LD) in the studied population. GBS SNPs discovered here were largely confined (~ 90%) to genic or gene-associated regions of the genome demonstrating the utility of using a methylation-sensitive restriction enzyme (*Pst*I) for library construction. As compared to Infinium array SNPs, GBS SNPs displayed enhanced effectiveness in estimating LD decay rates and discriminating population subgroups. GWAS using a combined set of 30,363 SNPs identified 189 unique QTL marker–trait associations (QTL-MTAs) covering all studied traits. The majority of the QTL-MTAs were from GBS SNPs potentially illustrating the effectiveness of marker-dense de novo genotyping platforms in overcoming ascertainment bias and providing a more accurate correction for different levels of relatedness in GWAS models. GWAS also detected QTL ‘hotspots’ for several traits at previously known as well as newly identified genomic locations. Due to the current study exploiting genome-wide genotyping and de novo SNP discovery simultaneously on a large tetraploid panel representing a greater diversity of the cultivated potato gene pool, the reported sequence-tagged MTAs are likely to have higher transferability across a wider range of potato germplasm and increased utility for expediting genomics-assisted breeding for the several complex traits studied.

**Supplementary Information:**

The online version contains supplementary material available at 10.1007/s00122-024-04651-8.

## Introduction

Potato (*Solanum tuberosum* L.) is one of the world’s most economically important food crops and holds major significance for future global food security. Despite its importance and intensive breeding efforts, genetic gains for yield and other complex traits have only been slowly accumulated, and old potato varieties are still widely used in commerce (Bachem et al. 2019; Douches et al. 1996; Piepho et al. 2014). Barriers to improvement are largely due to the complexities caused by autotetraploidy and a highly heterozygous potato genome. Constant progress in developing new and improved potato cultivars must be achieved for meeting the challenges posed by emerging disease threats, unpredictable and accelerating climatic changes, and rising demands for niche products due to expansion of markets in the fresh and the processing sectors. However, only a limited fraction of the available genetic diversity has so far been exploited for pursuing breeding objectives. Concerted research efforts are needed to screen the available genetic diversity for identifying novel and beneficial trait alleles targeting the manifold challenges faced by this key crop plant. Moreover, in order to realize the true potential of genomics-assisted breeding in potato the availability of cost-effective high-throughput genotyping methodologies is essential. The advent of next-generation sequencing (NGS) and high-throughput genotyping platforms coupled with the availability of whole-genome sequence data has brought a step change in the way potato genetic studies are conducted. Sequence-based genotyping (SBG) methods are now the preferred approach for high-throughput low-cost genotyping as compared to previously deployed SNP (single-nucleotide polymorphisms) array technologies due to the several advantages they provide. These include reduced cost, greater flexibility and scalability, and the capability to scan polymorphisms without prior knowledge, characteristic of an ‘open system’ genotyping platform (Sharma and Bryan 2017).

Although next-generation sequencing costs continue to decline, deploying whole-genome sequencing (WGS) for routine genotyping applications is still cost prohibitive, and not usually required for performing genetic analyses such as linkage mapping, genome-wide association studies (GWAS) and genomic selection. A more cost-effective form of SBG employs construction of NGS libraries using genome complexity reduction, and reduced representation mainly achieved through sequence capture or restriction enzyme-based methods. This facilitates interrogation of the same subset of the genome across the target population in a reproducible manner. Many variants of the restriction enzyme-based SBG technique exist, the two most commonly used are restriction-site associated DNA sequencing (RAD-Seq) (Baird et al. 2008) and genotyping-by-sequencing (GBS) (Elshire et al. 2011). GBS does not require DNA size fractionation and, thus, offers a simplified NGS library preparation procedure over RAD-seq. Restriction enzyme-based SBG provides several advantages viz. simultaneous marker discovery and genotyping; complexity reduction is easy, rapid, specific and highly reproducible; highly multiplexed and less expensive; reduced sample handling; and few PCR and purification steps (Elshire et al. 2011). However, use of GBS does raise certain issues which need careful consideration. GBS can be prone to ascertainment bias due to differential methylation of the restriction sites if methylation-sensitive restriction enzymes are used. This necessitates collection of experimental material from plants at synchronized growth stages and from similar tissue types to avoid variation arising due to differential methylation patterns. As any other NGS technique, GBS is also affected by potentially large amounts of missing data which can occur due to presence/absence variation, polymorphic/methylated restriction site, library complexity and poor read coverage in the affected regions.

GBS has been exploited in several crop plant species (Furuta et al. 2017; Labate et al. 2020; Medina et al. 2020; Nguyen et al. 2020; Poland et al. 2012; Rabbi et al. 2014; Salinas-Aponte et al. 2014; Willman et al. 2022; Zhebentyayeva et al. 2019) to perform genetic studies including GWAS, and reports in potato are also increasing in number (Bastien et al. 2018; Byrne et al. 2020; Diaz et al. 2021; Sverrisdottir et al. 2017). GWAS offer considerable advantages over use of biparental populations, such as enhanced mapping resolution, a larger number of traits that can be assessed in a single study, as well as increased robustness and transferability of GWAS QTL across a wider germplasm pool. Combining modern genetic methodologies such as GWAS with SBG approaches provide unprecedented advances in trait QTL detection and transitioning towards ‘next-generation’ potato breeding. Numerous studies have successfully reported application of GWAS in potato (Berdugo-Cely et al. 2017; Carley et al. 2017; D’hoop et al. 2008, 2014; Fischer et al. 2013; Kloosterman et al. 2013; Lindqvist-Kreuze et al. 2014; Rosyara et al. 2016; Schönhals et al. 2016; Sharma et al. 2018; van Eck et al. 2017; Vos et al. 2017). Reports involving other SBG approaches in potato, especially for low cost genome scanning, are also emerging (Leyva-Perez et al. 2022).

The current study reports the development of a robust GBS procedure in potato and demonstrates its efficacy in QTL detection using a diverse potato association panel. Sharma et al. (2018) used this panel of 341 clones to evaluate different GWAS models in tetraploid potato using the Infinium 8k Potato SNP Array (Felcher et al. 2012; Hamilton et al. 2011). Here, we report the results of a GWAS analysis on sixteen key tuber quality and production traits using GBS and Infinium SNPs including assessments of genetic diversity, population structure, and linkage disequilibrium. We also compare the results from the two contrasting genotyping approaches involving fixed (used by Sharma et al. (2018)) and flexible (adopted in the current study) genotyping platforms.

## Materials and methods

### Germplasm, field trials, phenotyping and trait data analyses

Field trials and phenotyping of the GWAS panel were conducted in 2012 and 2013 at two different sites (Cambridge and York, UK) and are described in detail in Sharma et al. (2018). The GWAS panel comprised 341 diverse tetraploid genotypes, including a set of 57 advanced breeding lines (Supplementary Table S1). The panel largely comprised European founder and cultivated germplasm, but it also contained 29 non-European cultivars. This paper reports findings for the following traits measured on this panel in replicated trials (two replicates for each environment), but not all traits were phenotyped in every environment (Supplementary Table S2): after cooking blackening (ACB: For each observation unit, five tubers steamed for 20 min, cut in half and each half assessed for darkening after 30 min; 1–9, 1 severe); average tuber weight (ATW); black dot (BLK); black scurf (BLS); tuber skin brightness (BRT: tuber skin texture; 1–9, 9 very smooth); common scab (CSC) using ADAS Key No. 2.3.1 (Anonymous 1976); tuber dry matter (DRM: percentage dry matter based on air-dried and water-immersed weights); tuber eye depth (EYE: 1–9; 1 deep, 9 shallow); tuber flesh colour (FSH: 1–9; 1 white, 9 deep yellow); height breadth ratio (HBR: measure of canopy architecture); tuber shape (SHP: 1–6, 1 round); tuber sprouting (SPR); tubers per stem (TPS); total tubers per plant (TTU); tuber shape uniformity (UNI: 1–9, 9 very uniform); tuber yield (YLD: mean kg/plot). Plant and stem counts were performed after full emergence and before canopy closure. For stem counts, branches from main stems were excluded and measurements were taken before the development of secondary stems. For HBR, foliage height (measured from the top of the ridge to the top of the canopy) and breadth (measured across the width of the canopy) measurements were taken when plants reached approximately 80% ground cover but before canopy closure. For DRM, ten tubers (55–65 mm diameter) were gently washed to remove soil and air-dried followed by percentage dry matter calculations (Weltech PW-2050, Weltech International Ltd, UK) using tuber weight measurements in air and water. SPR observations were using tubers (55–65 mm diameter) stored at 10 °C without any sprout suppressants. Data for the longest tuber sprout was recorded eight weeks after 95% of panel tubers have broken dormancy (i.e. sprout length > 3 mm). Each observation unit comprised sprout data from 6 tubers. Tuber storage and sprout measurements were conducted at the Sutton Bridge Crop Storage Research (SBCSR) facility, AHDB, UK. For BLS and CSC, the percentage of surface area affected by these diseases was recorded in 7 categories viz., 0–1, > 1–5, > 5–10, > 10–25, > 25–50, > 50–75 and > 75–100. A severity score for each plot was calculated by multiplying the number of tubers in each category by the mid-point value and dividing the sum of these values by the total number of tubers assessed. For BLK a similar approach was adopted but the percentage of the affected surface area was scored in 6 categories viz., 0–1, > 1–10, > 10–25, > 25–50, > 50–75 and > 75–100. To generate mean phenotypic values for each trait, genotype was modelled as a fixed effect while all other effects were treated as random. For each environment, the best linear unbiased estimates (BLUEs) for all traits were calculated using REML implemented in Genstat 20th edition (VSN International Limited, http://www.vsni.co.uk). These trait BLUEs were used in GWAS as well as all other analyses involving phenotypic values. Trait pairwise Pearson correlation coefficients were calculated using R package ggstatsplot (Patil 2021) deploying “Bonferroni” adjustment method for *p* values for multiple comparisons and significance level threshold set to 5%. Trait broad-sense heritabilities (*H*^2^) on plot basis were estimated using R package StageWise (Endelman 2023).

### Construction of genotyping-by-sequencing libraries and sequencing

Genomic DNA was extracted from young leaf tissue from individual field grown plants using the Qiagen DNeasy Plant Maxi Kit (Qiagen) and quantified using the Quant-iTTM PicoGreen® dsDNA Assay Kit (Invitrogen, San Diego, CA). Previously, the GWAS panel was genotyped using the Infinium 8k Potato SNP Array (Felcher et al. 2012; Hamilton et al. 2011) as detailed in Sharma et al. (2018). The current study extends the genetic characterization of the same GWAS panel using a genotyping-by-sequencing (GBS) procedure adapted from Poland et al. (2012). Different restriction enzyme (RE) combinations (*Pst*I, *Nsi*I and *Sbf*I as rare cutters; *Mse*I and *Bfa*I as frequent cutters) were evaluated for constructing GBS libraries using potato *cv*. Desiree as a test sample. Actual fragment size distribution from test GBS libraries, in combination with in silico restriction digestion prediction using RestrictionDigest tool (Wang et al. 2016), was assessed to select the optimum restriction enzyme pair for library construction. For constructing final GBS libraries, DNA samples (100 ng each) from the GWAS panel were subjected to double restriction enzyme digestion using the optimum RE pair (*Pst*I–*Mse*I, selection procedure described under “Results” section) followed by adapter ligation in a 24-plex multiplexing format. *Pst*I adapters were barcoded (designed using Deena Bioinformatics, Netherlands) whereas *Mse*I adapters were used as common adapters for all samples (Supplementary Table S3). The processed samples were pooled and PCR-amplified to obtain an NGS-ready library. Fragment size analysis and additional quality checks on GBS libraries were performed using the 2200 TapeStation system (Agilent Technologies). Each finished GBS library (16 in total) was sequenced on a single lane of Illumina HiSeq 2500 platform to generate 150 bp single-end sequence reads. Illumina sequencing was performed at Edinburgh Genomics, University of Edinburgh, UK.

### Read mapping, variant detection and genotype calling

Sequence data from all 16 GBS libraries were deconvoluted into single sample reads using GBS-SNP-CROP-v.4.1 (Melo et al. 2016; Melo and Hale 2019). Processed reads from each accession were quality trimmed using Trimmomatic (Bolger et al. 2014) and mapped onto the potato reference (the doubled monoploid potato *S. tuberosum* Group Phureja DM 1-3 516 R44; hereafter referred to as DM) genome version 6.1 (Pham et al. 2020; Potato Genome Sequencing Consortium 2011) using Bowtie2 (Langmead and Salzberg 2012) followed by variant discovery using HaplotypeCaller and performing joint genotyping across all samples simultaneously using GenotypeGVCF programmes available through GATK (DePristo et al. 2011; McKenna et al. 2010). Variants were filtered for genotype level read depth (DP > = 15), genotype quality (GQ > = 10) and QualByDepth (QD > = 2) , i.e. the QUAL score normalized by unfiltered read depth of variant samples. This was implemented using GATK VariantFiltration and SelectVariants tools. Functional annotation of GBS SNPs was performed using SNPeff (Cingolani et al. 2012).

### Infinium SNP array data analysis

Infinium 8k Potato SNP Array (Felcher et al. 2012; Hamilton et al. 2011) genotyping data, previously reported by Sharma et al. (2018), was reanalysed. SNP allelic dosages (genotypes) were called using fitPoly R package which was able to resolve genotypic classes for 6401 SNPs. SNP genomic coordinates were annotated according to DM assembly v6.1 (Pham et al. 2020).

### Genetic diversity and population structure

Population structure in the diversity panel was examined using two clustering methodologies viz., principal components analysis (PCA) and *k*-means clustering. PCA was performed over genomic relationship matrix implemented in the R package ASRgenomics and scree plot from the PCA analysis was used to examine germplasm diversity and infer the number of subpopulations (*Q*) in the association panel. *K*-means clustering was performed using ‘find.clusters’ function in adegenet (Jombart 2008; Jombart and Ahmed 2011) to identify the number of genotypic groups. The optimal number of *k*-means [equalling number of subpopulations (*Q*)] was determined by using the Bayesian information criterion (BIC) as a statistical measure of goodness of fit. The relationship between population groups was also assessed using DAPC (Discriminant analysis of Principal Components). The densities of individuals were plotted as a one-axis density plot (single discriminant function) to visualize the level of discrimination among population groups. A dendrogram heatmap of the genomic relationship matrix was used to visualize the genetic kinship among the genotypes included in the panel.

### Genome-wide association analysis

GWAS were performed using GWASpoly version 2.10 (Rosyara et al. 2016) employing additive, general, simplex dominance, duplex dominance, diplo-general and diplo-additive gene (genetic) action models as described in GWASpoly manual. Each genetic effect model was further evaluated using four different statistical models viz. (1) Naïve model, without controlling any confounding effects, (2) Kinship model, controlling just for individual relatedness (K), (3) population Structure model, controlling for population structure (*Q*) effects only, and (4) full model, accounting for *K* as well as *Q* confounding effects. All these four models are hereafter referred to as Naïve, *K*, *Q* and *QK* models, respectively. Within each genetic model, fitness of different statistical models was evaluated using Quantile–Quantile (*Q*–*Q*) plots of the observed versus expected − log_10_(*p*) values which should follow a uniform distribution under the null hypothesis. Models were ranked using genomic control inflation factor (*λ*_GC_) metric calculated as the median of the resulting Chi-squared test statistics divided by the expected median of the Chi-squared distribution. The LD-based Bonferroni-type multiple testing correction method “M.eff” (with genome-wide *α* = 0.05) was applied for establishing a *p* value (− log_10_(*p*)) detection threshold for statistical significance. The “M.eff” method in GWASpoly implements a Bonferroni-type correction using an effective number of markers that accounts for LD between markers (Moskvina and Schmidt 2008). For selecting a single marker per QTL, markers with scores above the set threshold for statistical significance were filtered to obtain the most significant marker–trait association (MTA) within a specified window set using the average extent of LD decay in the panel.

### Estimation of linkage disequilibrium (LD) decay

Whole chromosome-scale LD was estimated following the procedure as previously described (Sharma et al. 2018; Vos et al. 2017). Briefly, Pearson correlation coefficient (*r*^2^) was used to calculate correlations between marker-pairs using SNP dosage scores (0–4) and LD was estimated based on marker pairs located within each of the 12 individual chromosomes. Extent of LD decay was estimated by implementing quantile regression (R package ‘quantreg’; Koenker 2017) on the 90th percentile. From the fitted regression, two LD estimators were obtained, viz. LD_1/2max,90_ and LD_1/10,90_, denoting the distances (in Mb) at which LD equals one-half of its maximum fitted *r*^2^ value (*r*^2^_max,90_) and where *r*^2^ reaches one-tenth on the 90th percentile, respectively. LD estimates, for both datasets (GBS and Infinium SNP array), were computed with SNPs used for performing GWAS.

## Results

### Optimization of restriction enzyme combination for constructing GBS libraries

**Fig. 1:**
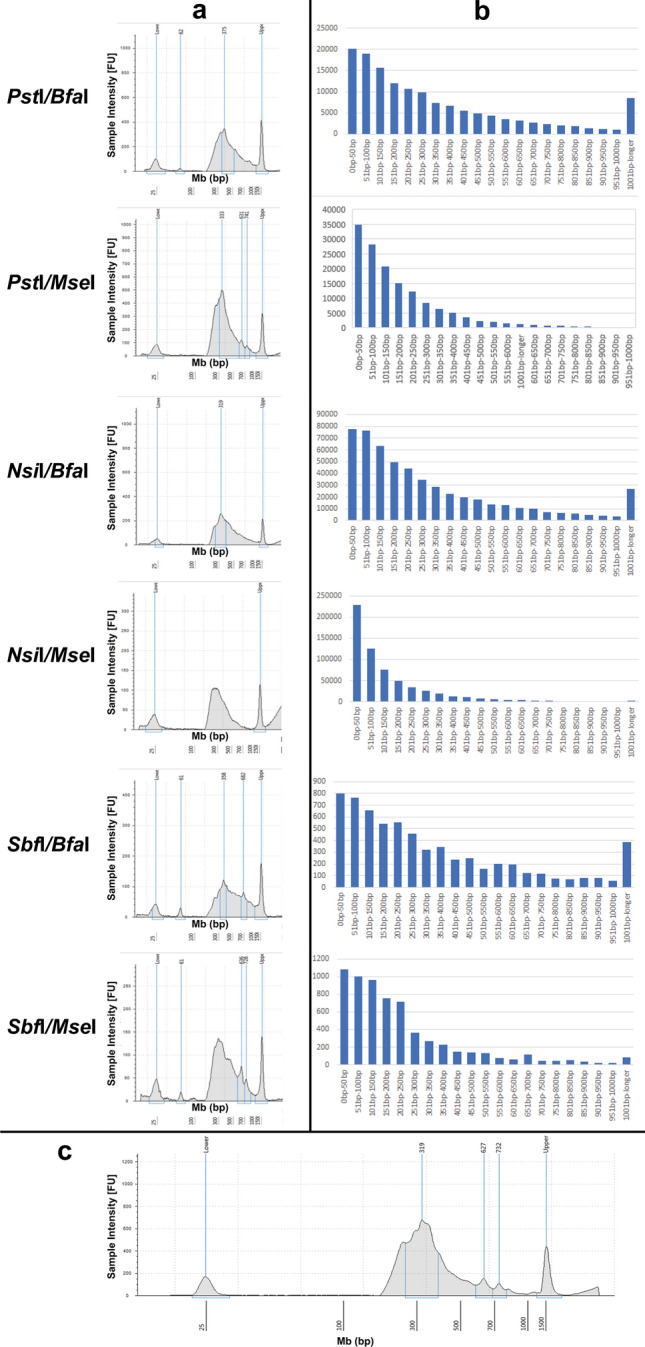
Illustration of genomic DNA fragment size distribution from: (**a**) test GBS libraries obtained using six restriction enzyme pairs, (**b**) in silico restriction digestion prediction using six restriction enzyme pairs and, (**c**) representative ‘Illumina sequencing ready’ 24-plex GBS library constructed using *Pst*I/*Mse*I

A total of six restriction enzyme (RE) pairs, comprising one ‘rare’ cutter (*Pst*I: methylation-sensitive 6-base cutter, *Nsi*I: 6-base cutter, or *Sbf*I: 8-base cutter) and one ‘frequent’ cutter (*Mse*I: 4-base cutter or *Bfa*I: 4-base cutter), were examined for their suitability to construct potato GBS libraries. The fragment size distribution from test GBS libraries obtained using these six RE pairs as well as that predicted from in silico restriction digestion is illustrated in Fig. 1a, b. The number of fragments (GBS-tags) generated using *Nsi*I combination pairs was relatively high especially in the smaller fragment-size range (0–100 bp). The overall number of fragments generated using *Sbf*I combination pairs was low considering it being an 8-base cutter. As compared to the former two REs, number and distribution of GBS-tags generated using *Pst*I combination pairs was found to be in the optimum fragment size and number range for constructing GBS libraries. The propensity of GBS tags in the size-range beyond that is suitable (i.e. 200–500 bp, excluding adapters (Quail et al. 2009)) for effective cluster amplification during standard Illumina short-read sequencing was higher in libraries generated using *Bfa*I in combination with all three rare cutters as compared to those obtained using *Mse*I. Considering the number and fragment-size distribution obtained from test libraries, a combination of ‘*Pst*I/*Mse*I’ restriction enzymes was chosen to construct final GBS libraries. A 24-plex multiplexing regime was followed requiring the generation of sixteen GBS libraries containing 361 tetraploid clones (341 net samples, 15 Desiree controls and 5 samples with identity issues later removed from the study), 22 diploid clones (including 16 DM control samples) and one hexaploid genotype (Supplementary Table S4). Figure 1c shows fragment size distribution for one of these ‘Illumina sequencing ready’ 24-plex GBS libraries constructed here.

### Genome-wide SNP discovery using genotyping-by-sequencing

Sequencing on an Illumina HiSeq 2500 yielded ~ 1889 million reads in total averaging 118 million reads per GBS library. Sample deconvolution using GBS-SNP-CROP-v.4.1 (Melo et al. 2016; Melo and Hale 2019) assigned an average of 103.1 million net usable reads per GBS library (~ 4.3 million per sample) displaying a range of 82.1 (GBS Lib 07) to 131.5 (GBS Lib 09) million reads per library (Supplementary Table S5). Using the read mapping and variant discovery procedure, as described in the “Materials and methods” section, a total of 210,614 polymorphisms (204,397 SNPs and 6217 indels) were initially identified across all samples included in the GBS panel. Of these variants, 192,282 SNPs were identified for the 341-clone tetraploid set (hereafter referred to as ‘total-tetra-set’) and 63,743 SNPs were obtained in the non-tetraploid (2× and 6×) genotype set (not part of the current study) indicating an average of one GBS SNP per ~ 3.9 kb and ~ 11.6 kb for both germplasm panels, respectively. The genomic locations of ‘total-tetra-set’ SNPs intersected 24,181 genes (DMv6.1, data not shown).

**Fig. 2:**
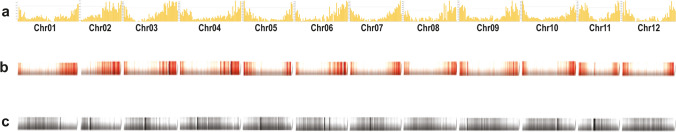
Illustration of genome-wide distribution (per 1 Mb) of (**a**) GBS SNPs, (**b**) gene density, and (**c**) repeat regions density

Filtering of ‘total-tetra-set’ markers for low sample genotype level read depth (DP < 15), low genotype quality (GQ < 10), low quality-by-depth (QD < 2.0), variant type (only biallelic SNPs) and excluding non-variants, yielded 116,048 high-quality biallelic single-nucleotide variants (SNVs). Of these, 99.95% SNPs (115,985) were physically mapped across 12 potato chromosomes with an average of one SNP per ~ 6.4 kb. The highest and lowest marker rates were observed on chromosome 2 and chromosome 10 with an average of one SNP per ~ 4.0 kb and ~ 8.8 kb, respectively. The remaining 63 SNPs were physically mapped on unanchored superscaffolds (chromosome 0) of the potato genome. Figure 2 displays genome-wide SNP density plot and the distribution of SNPs by chromosomes is given in Table 1. The panel showed a Ts/Tv (Transitions/Transversions) ratio of 1.86 for the observed SNPs while the missense-to-silent ratio for ‘total-tetra-set’ SNPs was 0.85 (missense: 45.86%; silent: 53.74%; nonsense: 0.41%). The percentage of SNPs with high, low, moderate and modifier impact categories was 0.18, 15.22, 12.11 and 72.50, respectively. The filtered ‘total-tetra-set’ SNPs overlapped 19,998 genes, and the list of these genes along with SNP count affecting each gene and their respective impact categories is provided in Supplementary Table S6. The number of SNPs in different effect-type categories and regions is provided in Table 2.

**Table 1:** GBS SNP variant rate (average genomic distance per SNP) per chromosome

Chromosome	Length (bp)	SNP count	SNP rate (bp)
1	8,85,91,686	15,190	5832
2	4,61,02,915	11,606	3972
3	6,07,07,570	11,186	5427
4	6,92,36,331	10,684	6480
5	5,55,99,697	7786	7140
6	5,90,91,578	10,161	5815
7	5,76,39,317	8985	6415
8	5,92,26,000	8056	7351
9	6,76,00,300	9161	7379
10	6,10,44,151	6903	8843
11	4,67,77,387	8523	5488
12	5,96,70,755	7744	7705
0	1,02,97,348	63	1,63,449
Total	74,15,85,035	1,16,048	6390

**Table 2:** Details of GBS SNP effects by type and region

Effect type	SNP count	Percent^a^
3_prime_UTR_variant	3849	1.89
5_prime_UTR_premature_start_codon_gain_variant	172	0.08
5_prime_UTR_variant	1246	0.61
downstream_gene_variant	52,298	25.66
initiator_codon_variant	2	0.00
intergenic_region	20,860	10.24
intron_variant	37,281	18.29
missense_variant	24,376	11.96
splice_acceptor_variant	44	0.02
splice_donor_variant	50	0.03
splice_region_variant	2430	1.19
start_lost	17	0.01
stop_gained	216	0.11
stop_lost	29	0.01
stop_retained_variant	26	0.01
synonymous_variant	28,595	14.03
upstream_gene_variant	32,301	15.85

Effect region	SNP count	Percent^b^
DOWNSTREAM	52,298	25.98
EXON	52,915	26.29
INTERGENIC	20,860	10.36
INTRON	35,387	17.58
SPLICE_SITE_ACCEPTOR	44	0.02
SPLICE_SITE_DONOR	50	0.03
SPLICE_SITE_REGION	2181	1.08
UPSTREAM	32,301	16.05
UTR_3_PRIME	3849	1.91
UTR_5_PRIME	1418	0.70

^a^Percent SNPs out of the total SNP count for all ‘effect type’ categories

^b^Percent SNPs out of the total SNP count for all ‘effect region’ categories

The SNP dataset was further filtered for 290 tetraploid clones (hereafter referred to as ‘pheno-tetra-set’) included in trait phenotyping. All genetic analyses were performed using ‘pheno-tetra-set’ biallelic SNPs after removing monomorphic variants and those displaying higher (> 20%) missing data, lower (< 1%) minor allele frequencies (MAF) and higher (> 0.98) maximum genotype frequency. The procedure yielded a robust set of 24,518 SNPs. Genotypes were called in true tetraploid allele dosage levels (0, 1, 2, 3 and 4) using R package GWASpoly (Rosyara et al. 2016).

### Genetic characterization and population structure analysis

**Fig. 3:**
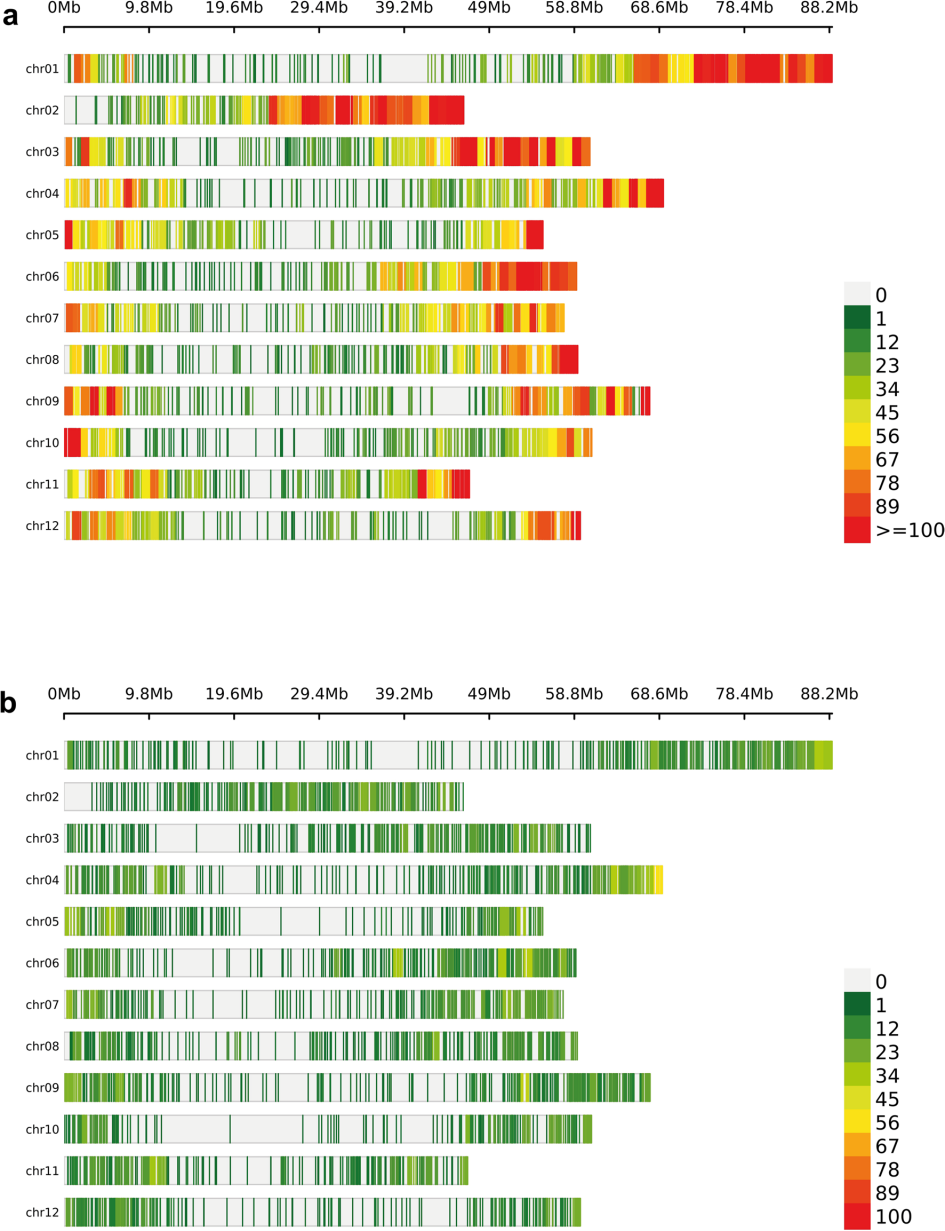
Distribution of (**a**) GBS and (**b**) Infinium array SNPs used in all genetic analyses. Bin size = 1 Mb

**Fig. 4:**
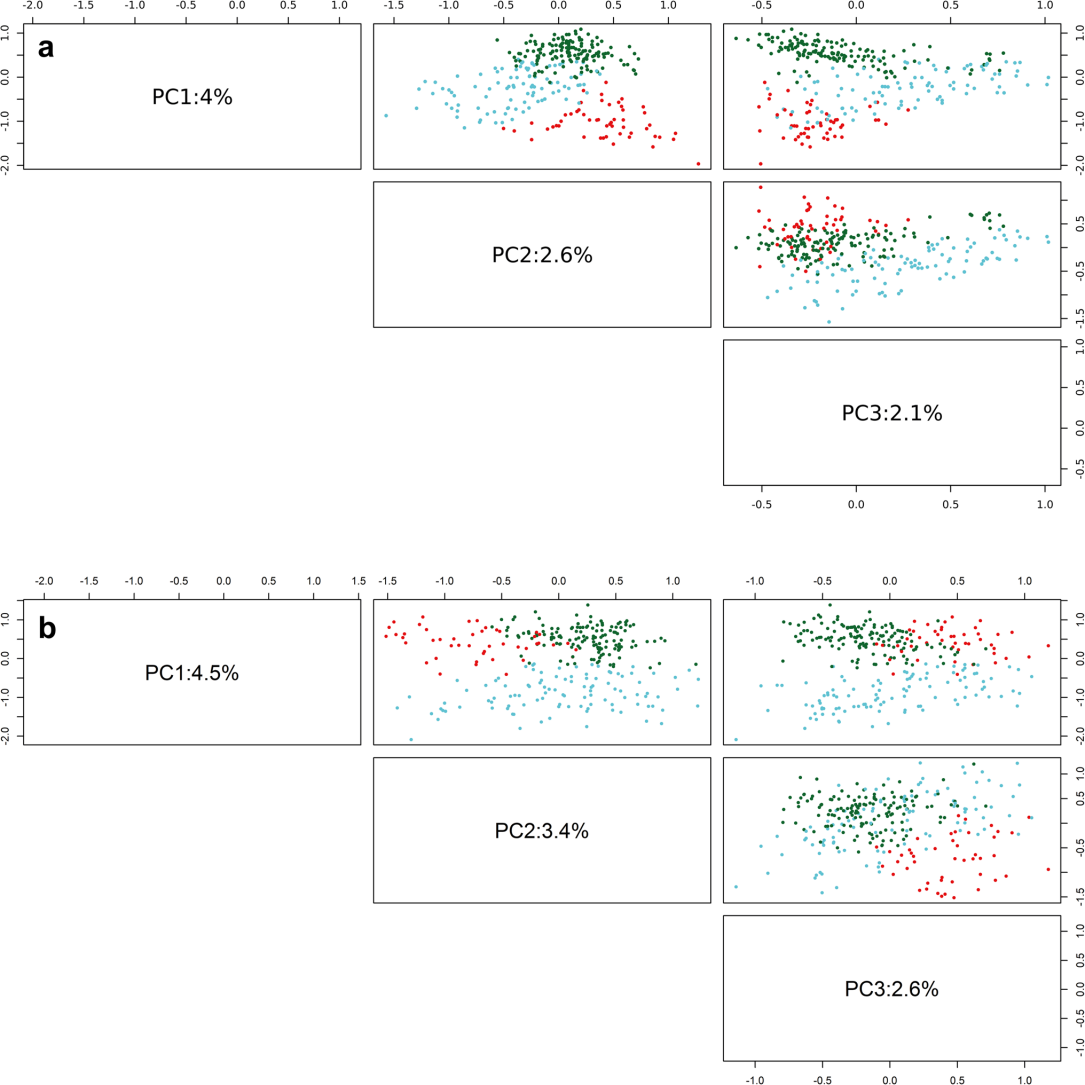
Principal component analysis of 290 tetraploid potato GWAS panel clones using (**a**) 24,518 GBS SNPs and (**b**) 5845 Infinium array SNPs. The individual clones are coloured on the basis of their membership to subpopulations (clusters) detailed in Supplementary Table S7 (GBS: Cluster1 = red, Cluster2 = green, Cluster3 = cyan; Infinium: Cluster1 = green, Cluster2 = red, Cluster3 = cyan)

**Fig. 5:**
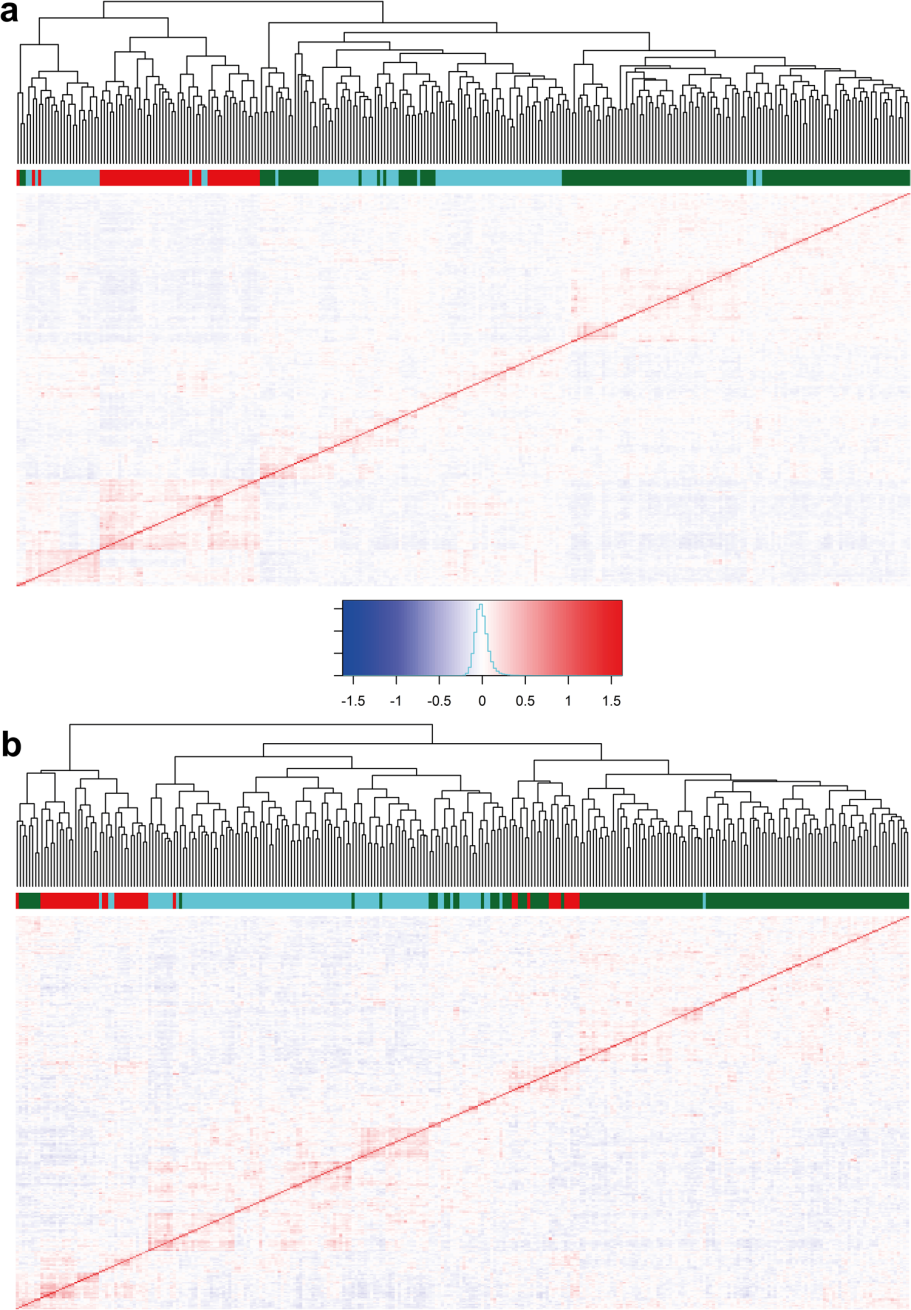
Heatmap displaying relationships among 290 tetraploid potato GWAS panel clones using (**a**) 24,518 GBS SNPs and (**b**) 5845 Infinium array SNPs. The red diagonal represents perfect relationship of each line with itself; the symmetric off-diagonal elements represent relationship for pairs of lines where warmer colour indicates a positive relationship and colder colour denotes a negative relationship. The colours in the horizontal bar below dendrogram depict clones’ membership to subpopulations (clusters) as detailed in Supplementary Table S7 (GBS: Cluster1 = red, Cluster2 = green, Cluster3 = cyan; Infinium: Cluster1 = green, Cluster2 = red, Cluster3 = cyan)

**Fig. 6:**
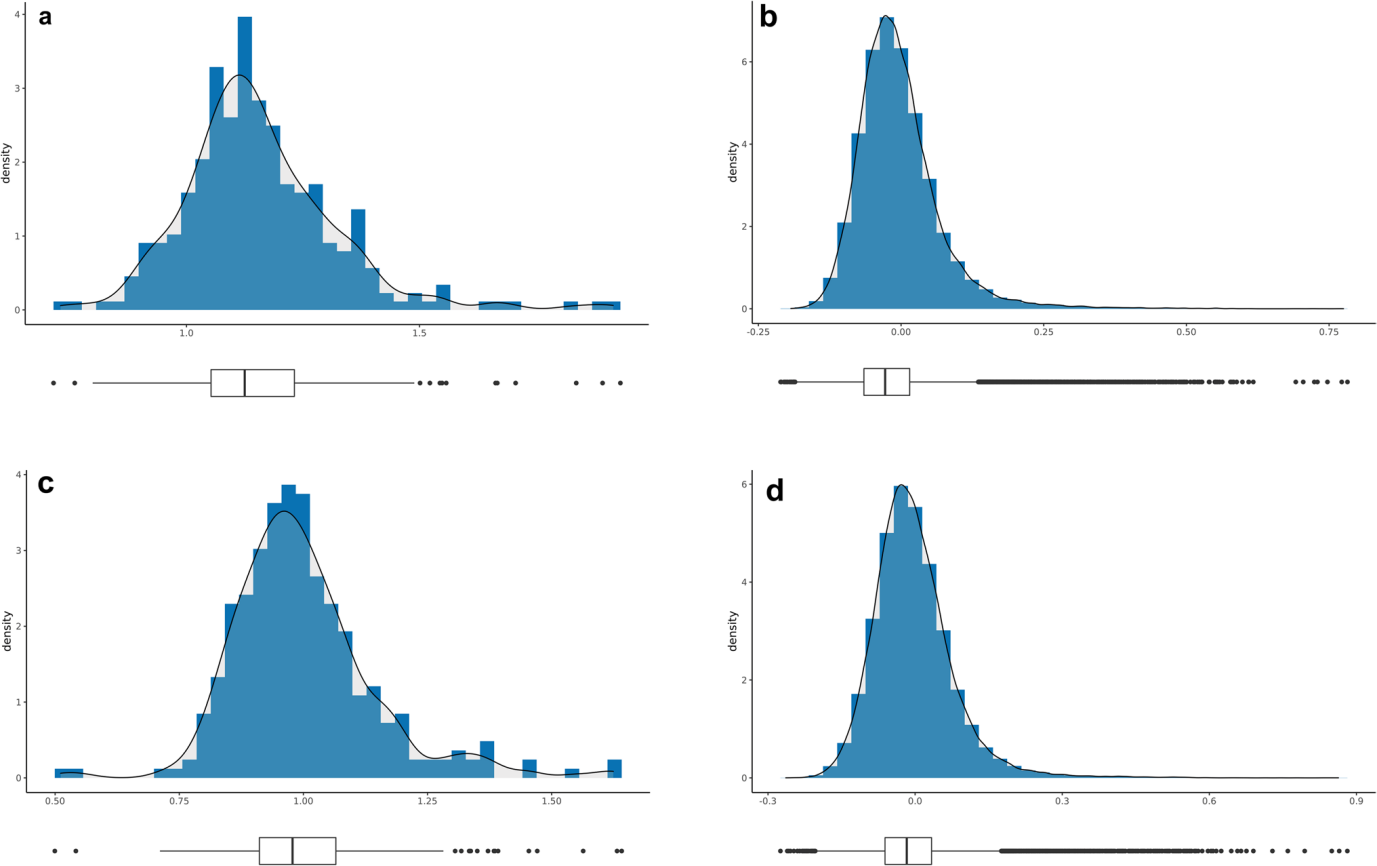
Histogram plots for the diagonal and off-diagonal values of the genomic relationship matrix using GBS (24,518 SNPs; **a**, diagonal values; **b**, off-diagonal values) and Infinium array (5845 SNPs; **c**, diagonal values; **d**, off-diagonal values) datasets

The GWAS panel (pheno-tetra-set) was genetically characterized using SNPs detected by GBS in the current study as well as using the Infinium 8k Potato SNP Array (Felcher et al. 2012; Hamilton et al. 2011) previously reported by Sharma et al. (2018), as described in the “Materials and methods” section. Processing of Infinium array SNPs using the filtering criteria as described in the preceding section for GBS variants provided an effective set of 5845 SNPs. Figure 3 displays pattern of distribution and marker density for 24,518 GBS and 5845 Infinium SNPs used for all subsequent analyses. Population structure was analysed using principal component analysis (PCA) and *K*-means clustering. Assessment of the scree plot (point of inflection/elbow junction) from the PCA analysis indicated the presence of three subpopulations (*Q*) within the association panel (Supplementary Fig. S1). This was further corroborated by the cluster detection based on Bayesian information criterion (BIC) which supported the presence of three clusters as ‘*k* = 3’ was within the shallow minimum of the BIC ‘goodness of fit’ curve (Supplementary Fig. S2) indicating three clusters are most optimum for classifying genotypes in the panel. The pattern of population stratification and the percentage of the genetic variation accounted by the first three components of the PCA using GBS and Infinium array SNPs is illustrated in Fig. 4. The relationship between subpopulation groups was also assessed using DAPC (Discriminant analysis of Principal Components DAPC). Visualizing densities of individuals on a single-axis density plot (single discriminant function) revealed enhanced level of discrimination among the three subpopulation groups by using GBS SNPs than that achieved by the Infinium SNPs (Supplementary Fig. S3). The *K*-means-based cluster (subpopulations) membership for the GWAS panel clones along with their market segment, year of release and country of origin details is provided in Supplementary Table S7. The panel subpopulations did not display any correspondence with clone market segment categories (Supplementary Table S7). However, as reported by Sharma et al. (2018), ‘country of origin’ did show some association with PCA subpopulations where cluster 1 predominantly contain modern UK cultivars while the other two clusters include old and modern cultivars from the UK, European and non-European countries. The genetic kinship among the genotypes included in the GWAS panel was visualized as a dendrogram heatmap of the genomic relationship matrix (Fig. 5). Figure 6 displays histogram plots for the diagonal and off-diagonal values of the genomic relationship matrix using GBS and Infinium SNPs. The diagonal histogram plot for both SNP datasets is centred around one (GBS: 1.16; Infinium: 1.00) as expected for non-inbred individuals. Similarly, the off-diagonal histogram plot for both SNP datasets is centred around zero (GBS: − 0.0040; Infinium: − 0.0034) as presumed for the unrelated individuals.

### Linkage disequilibrium analysis

**Fig. 7:**
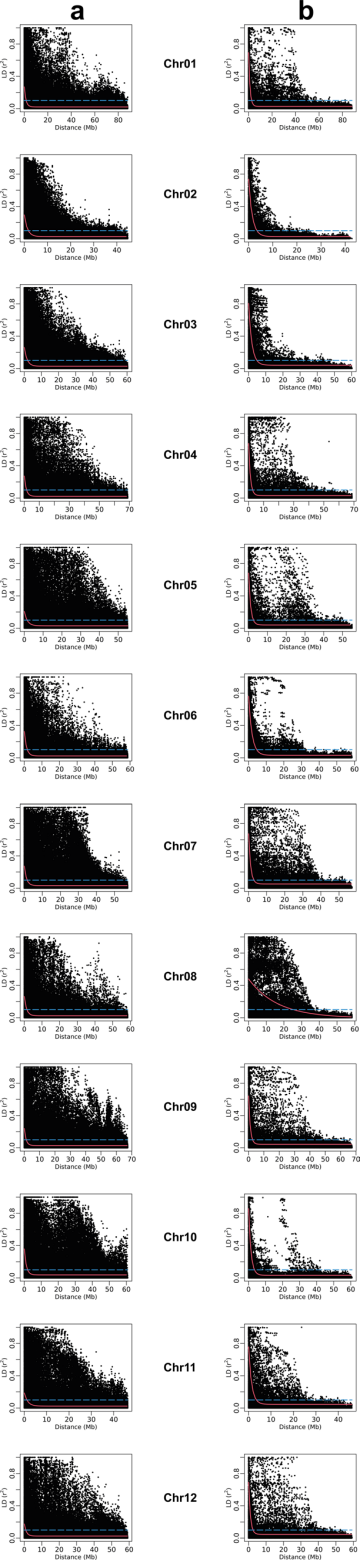
Linkage disequilibrium (LD) measure *r*^2^ in the potato association panel plotted versus the physical map distance (Mb) between pairs of SNPs (**a**, GBS SNPs; **b**, Infinium array SNPs) located on the whole chromosomal region for all 12 chromosomes. The trend line of the nonlinear quantile regression of *r*^2^ (90th percentile) versus the physical map distance between the SNP markers is illustrated in red while dashed blue line depicts the standard LD decay threshold (*r*^2^ = 0.1)

Linkage disequilibrium in the GWAS panel was assessed using Pearson’s *r*^2^ statistic using pairwise combinations of SNPs present across all 12 chromosomes. The extent of LD decay was estimated at the whole chromosome level using two LD estimators, viz. LD_1/2max,90_ and LD_1/10,90_, denoting the distances (in Mb) at which LD equals one-half of its maximum fitted *r*^2^ value (*r*^2^_max,90_) and where *r*^2^ reaches one-tenth on the 90th percentile, respectively. The analysis was performed using GBS as well as 8k Infinium array SNPs. The extent of LD decay in the GWAS panel using both SNP datasets is illustrated in Fig. 7, and the *r*^2^_max,90_, LD_1/2max,90_ and LD_1/10,90_ estimates from the two datasets are provided in Table 3.

**Table 3:** Extent of LD decay in the GWAS panel estimated using GBS and Infinium array SNPs at whole chromosome level

Chromosome	GBS SNPs			Infinium array SNPs		
	*r*^2^_max,90_	LD_1/2max,90_	LD_1/10,90_	*r*^2^_max,90_	LD_1/2max,90_	LD_1/10,90_
1	0.27	1.10	1.61	0.69	0.88	2.64
2	0.29	1.08	1.74	0.74	1.11	3.44
3	0.26	1.35	1.93	0.81	1.42	4.84
4	0.27	1.02	1.52	0.67	0.89	2.66
5	0.21	1.42	1.50	0.68	0.82	2.55
6	0.33	0.97	1.72	0.76	1.44	4.60
7	0.27	1.05	1.59	0.67	0.88	2.88
8	0.26	1.03	1.48	0.48	11.27	25.29
9	0.24	0.97	1.24	0.64	0.84	2.59
10	0.36	0.96	1.92	0.86	1.01	3.46
11	0.19	1.27	1.13	0.76	1.02	3.47
12	0.17	1.02	0.80	0.69	0.78	2.52
Average	0.26	1.10	1.52	0.70	1.86	5.08

*r*^2^_max,90_: maximum Pearson correlation coefficient (*r*^2^) achieved in the 90th percentile

LD_1/2max,90_: physical distance (Mb) at which LD has decayed to half its maximum *r*^2^ value in the 90th percentile

LD_1/10,90_: physical distance (Mb) at which LD has decayed to *r*^2^ = 1/10 in the 90th percentile

The average LD_1/2max,90_ and LD_1/10,90_ estimates for the panel using GBS SNPs were 1.10 Mb and 1.52 Mb, respectively, while the same estimates using Infinium SNPs were 1.86 Mb and 5.08 Mb, respectively (Table 3). LD_1/10,90_ metric displayed a faster LD decay for all chromosomes using GBS SNPs as compared to the Infinium array SNPs, however, the trend using LD_1/2max,90_ estimates showed mixed patterns where GBS SNPs revealed faster LD decay for five chromosomes (2, 3, 6, 8 and 10) and Infinium SNPs for seven chromosomes (1, 4, 5, 7, 9, 11 and 12). The LD estimates using GBS and Infinium SNPs differed to a maximum extent by 1.7× (LD_1/2max,90_; chromosome 5) and 3.1× (LD_1/10,90_; chromosome 12) among all chromosomes except chromosome 8 where the LD decay rates using GBS SNPs were significantly sharper (10.9× and 17.1× for LD_1/2max,90_ and LD_1/10,90_, respectively) than those displayed by the Infinium SNPs.

### Phenotyping

The association panel was phenotyped for sixteen production, agronomic and processing traits as described in the “Materials and methods” section. The GWAS panel displays a broad range of phenotypic diversity for most traits, and largely the residuals for all traits were normally distributed (Supplementary Fig. S4). As the panel largely contains cultivated potato varieties, for some commercially important traits the phenotypic scores are biased due to selection for the more favourable scores; nevertheless, the variation was sufficient for performing genetic analysis on these traits.

**Fig. 8:**
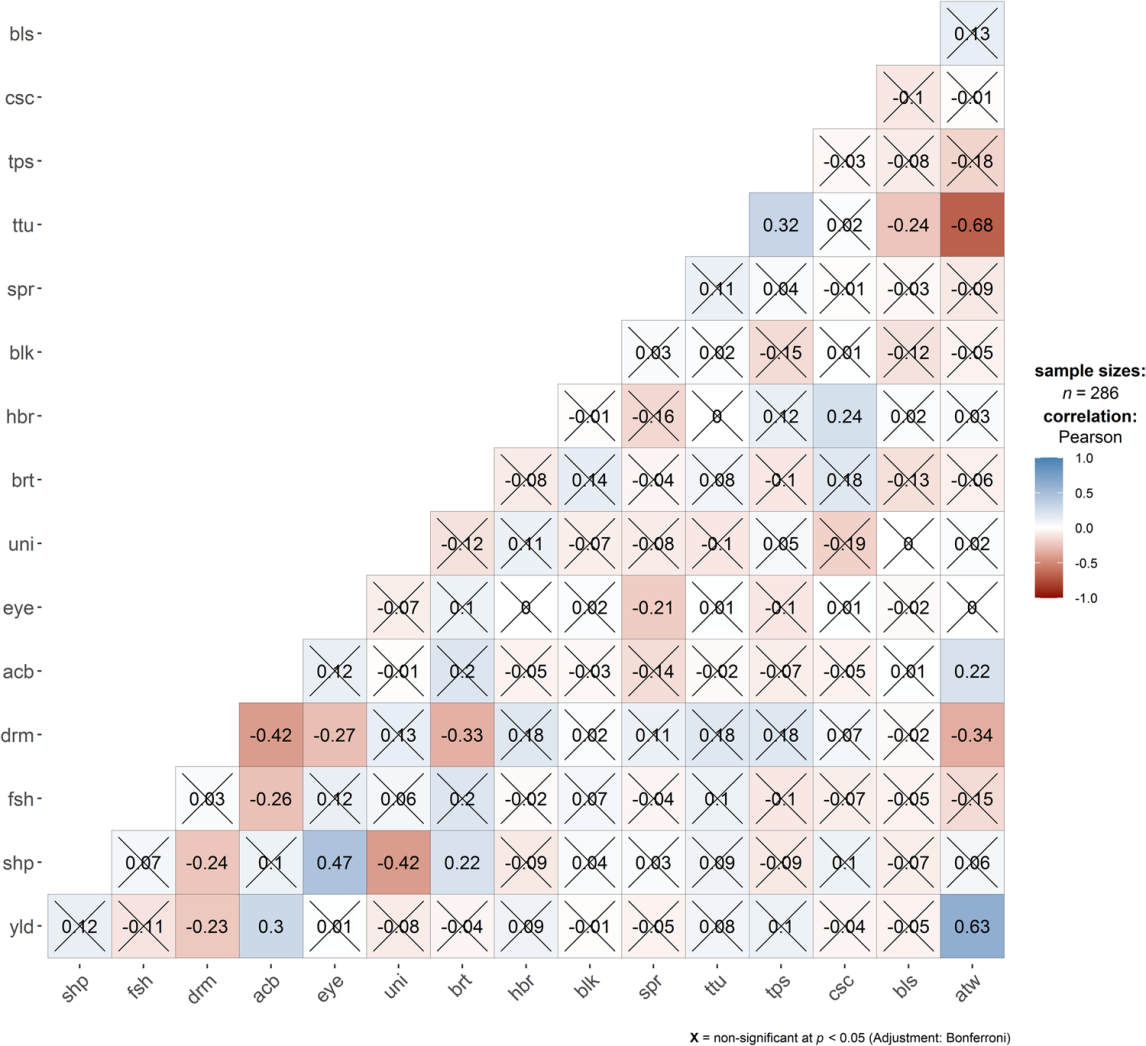
Correlation heatmap of all 16 traits included in the study

Average broad-sense heritability (*H*^2^, plot basis) estimates showed a wide variation (0.18–0.91), but for most traits the *H*^2^ values were in the moderate-to-high range (0.4–0.8), with three (black scurf, after cooking blackening and tuber uniformity) and four (tuber shape, average tuber weight, tuber dry matter and tuber flesh colour) traits displaying low and very high heritabilities, respectively (Table 4). Significant correlations were observed between some traits, while correlations between most trait-pair combinations were non-significant (Fig. 8).

**Table 4:** Trait broad-sense heritability (*H*^2^, plot basis) estimates

Trait	CAMBRIDGE_2012	CAMBRIDGE_2013	YORK_2012	YORK_2013	Average *H*^2^
acb	0.23	0.26	0.37	0.18	0.26
atw	0.71	–	0.88	0.87	0.82
blk	0.61	0.24	0.43	0.45	0.43
bls	–	0.20	–	0.15	0.18
brt	0.77	0.71	0.80	0.77	0.76
csc	–	0.32	0.54	0.51	0.46
drm	0.88	0.74	0.90	0.82	0.84
eye	0.51	0.58	0.57	0.63	0.57
fsh	0.91	0.91	0.91	0.89	0.91
hbr	0.59	–	0.35	0.46	0.47
shp	0.81	0.78	0.81	0.83	0.81
spr	0.72	–	0.73	–	0.73
tps	0.63	–	0.65	–	0.64
ttu	0.73	–	0.72	0.81	0.75
uni	0.30	–	0.30	0.37	0.32
yld	0.81	–	0.71	0.73	0.75

### GWAS analyses

GWAS analyses utilized phenotypic and genotypic data from ‘pheno-tetra-set’ comprising 290 tetraploid clones. For each trait, GWAS were conducted using (a) only GBS SNPs (24,518), (b) only Infinium SNPs (5845), and (c) a combined (GBS and Infinium) marker set (30,363 SNPs); each GWAS-type hereafter referred to as GBS-GWAS, Infinium-GWAS and GBS–Infinium–GWAS, respectively. All GWAS analyses were performed using the autotetraploid genotype model (coding dosage level for alternate allele as 0, 1, 2, 3 or 4 for each biallelic SNP), and employing all six (additive, general, simplex dominance, duplex dominance, diplo-general and diplo-additive) gene action models as described in the R package GWASpoly manual. Each gene action model was further evaluated using Naïve, *K*, *Q* and *QK* models, and fitness of these statistical models was evaluated using *Q*–*Q* plots and genomic control inflation factor (*λ*_GC_) metric as described in the “Materials and methods” section. First three principal components (PC) were deemed relevant for describing population structure based on the point of inflection observed in the PCA scree plot (Supplementary Fig. S1) and BIC ‘cluster detection’ goodness of fit curve (Supplementary Fig. S2); and were included to form the *Q* matrix in *Q* and *QK* GWAS models for controlling population confounding effects. The marker–trait associations (MTAs) were declared statistically significant on the basis of *p* value (− log10(*p*)) detection threshold established using the “M.eff” method (implemented in GWASpoly) to control the genome-wide false positive rate (*α* = 0.05).

**Fig. 9:**
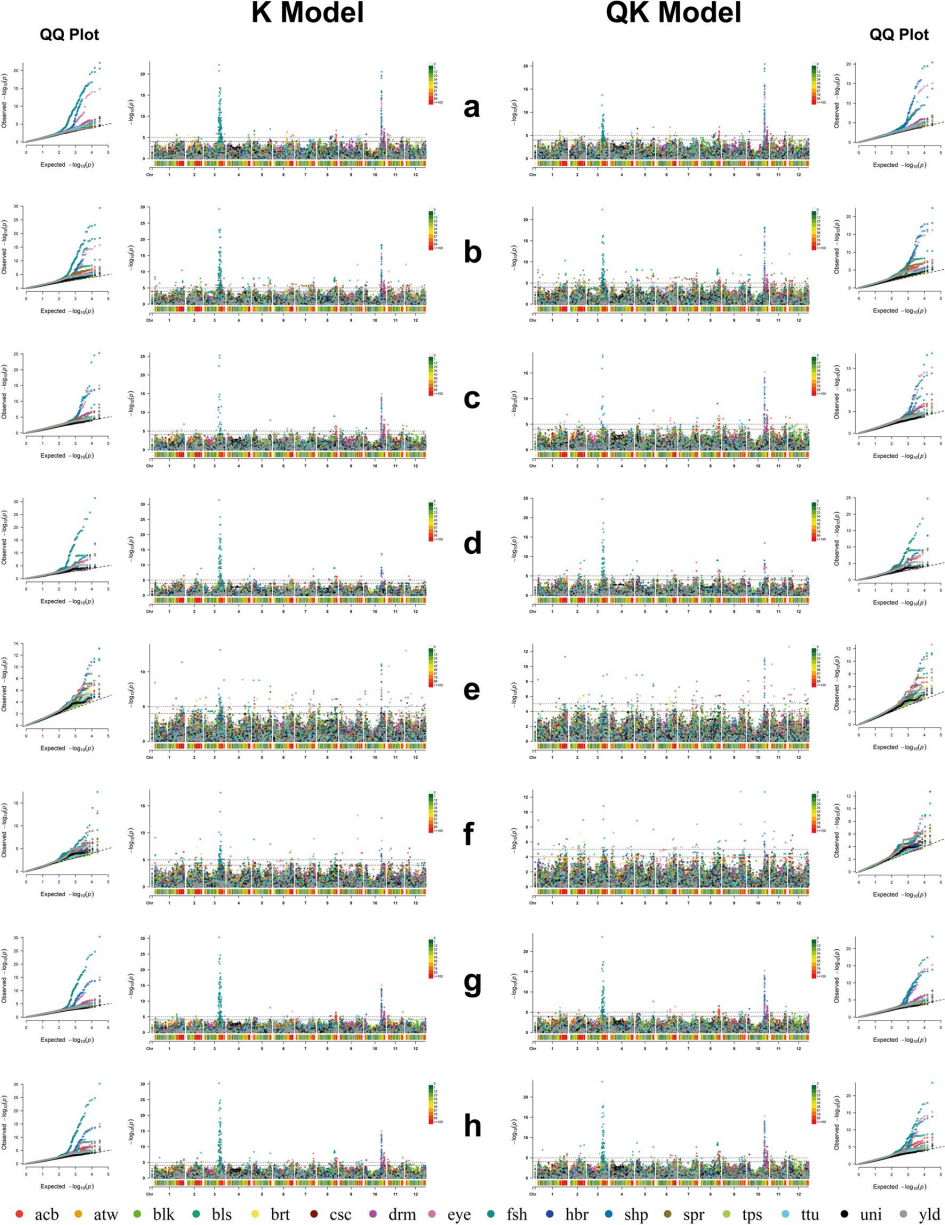
Combined Manhattan and QQ plots from K and QK GWAS statistical models for all 16 traits using different gene action models (**a**, additive; **b**, general; **c**, simplex dominance-alt; **d**, simplex dominance-ref; **e**, duplex dominance-alt; **f**, duplex dominance-ref; **g**, diplo-additive; **h**, diplo-general). GWAS main significance threshold (upper black dashed line) and suggestive significance threshold (lower black dashed line) are set to 1e-5 and 1e-4 −log10(*p*) values, respectively. Manhattan plots using significance thresholds obtained separately for each trait using Bonferroni-type multiple testing correction method “M.eff” (with genome-wide *α* = 0.05) are shown in Supplementary Fig. S6. The heatmap below Manhattan plots illustrate SNP density (bin size = 1 Mb)

**Fig. 10:**
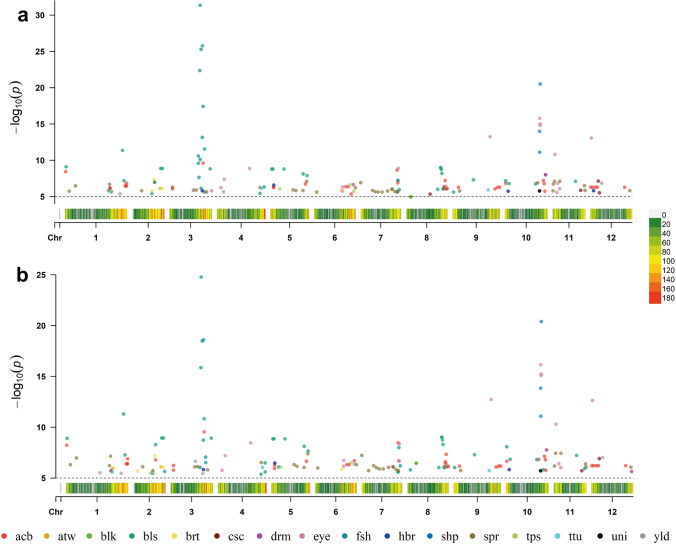
Graphical illustration of the non-redundant set of unique QTL-MTAs (**a**, K models; **b**, QK models) listed in Table 5. Black dashed line depicts GWAS main significance threshold set to 1e-5 − log10(*p*) value, while actual significance thresholds obtained separately for each trait MTA using Bonferroni-type multiple testing correction method “M.eff” (with genome-wide *α* = 0.05) are provided in Table 5. The chromosome heatmaps below each plot illustrate SNP density (bin size = 1 Mb)

For simplification and brevity, we focus here predominantly on results from the GBS–Infinium–GWAS analysis. Supplementary Fig. S5 displays *Q*–*Q* plots comparing the inflation of *p* values for the four principal GWAS models deployed for each genetic (gene action) model for all 16 traits, and Supplementary Table S8 presents genomic control inflation factor (*λ*_GC_) values for each trait and ‘GWAS genetic model × statistical model’ combination. Supplementary Fig. S6 illustrates individual trait Manhattan plots from all GWAS and gene action models analysed here while the combined Manhattan plots from *K* and *QK* models for all 16 traits and each gene action model are presented in Fig. 9. The initial GWAS scan resulted in a non-redundant set of 1181 significant MTAs from *K* and *QK* models (redundancy for same SNP MTAs appearing for more than one trait was not removed) covering all traits assessed in the study including MTAs appearing with multiple gene action models for the same SNP (Supplementary Table S9a). These initial MTAs were filtered for selecting the most significant marker per LD-based moving window scan and a single MTA per LD-based window (genomic interval) was retained (hereafter referred to as QTL–MTA) for each gene action model GWAS. The window-size (1.52 Mb) for LD-based scan was set using the average extent of LD decay (average LD_1/10,90_ distance derived using GBS SNPs) in the panel. The most significant QTL-MTAs from K and QK models for each GWAS-type and trait are listed in Supplementary Table S9b, where all QTL-MTAs for SNPs appearing with more than one gene action model are included. Table 5 lists a non-redundant set of 189 unique QTL-MTAs where only a single QTL–MTA, for those appearing with multiple gene action models, with the highest significance value was retained and forms the basis of GWAS results presented in this study. Graphical representation of MTAs listed in Table 5 is presented in Fig. 10.

**Table 5:** List of unique QTL-marker-trait associations (QTL-MTAs) following LD-based filtering and retaining MTAs with most significant *p* value per LD window interval. For QTL-MTA SNPs appearing with multiple gene action models, only the SNP and gene action model combination giving the most significant *p* value was kept

Trait	Marker^a^	Chrom	Position	Model	Threshold	Score (*K*)	Effect (*K*)	Score (*QK*)	Effect (*QK*)
acb	solcap_snp_c2_56714	chr01	1083841	2-dom-alt	5.55	8.43	− 0.817	8.23	− 0.805
acb	chr01_86122529	chr01	86122529	2-dom-ref	5.45	6.45	− 1.126	6.39	− 1.118
acb	chr01_87668102	chr01	87668102	1-dom-ref	5.31	6.45	− 1.126	6.39	− 1.118
acb	chr01_87696636	chr01	87696636	2-dom-ref	5.45	6.45	− 1.126	6.39	− 1.118
acb	chr01_88032313	chr01	88032313	1-dom-alt	5.55	6.82	0.693	6.88	0.695
acb	chr02_32301311	chr02	32301311	1-dom-ref	5.31	6.97	− 0.980	6.79	− 0.966
acb	chr03_4471195	chr03	4471195	2-dom-alt	5.55	6.29	− 1.108	6.23	− 1.099
acb	chr03_4473984	chr03	4473984	general	5.57	6.07		5.77	
acb	solcap_snp_c1_6348	chr03	48304511	1-dom-ref	5.31	9.61	− 1.049	9.54	− 1.043
acb	chr05_4616950	chr05	4616950	general	5.57	6.24		5.95	
acb	chr05_4664299	chr05	4664299	2-dom-alt	5.55	6.3	− 1.114	6.27	− 1.108
acb	chr05_50172505	chr05	50172505	general	5.57	6.73		6.64	
acb	chr06_45447204	chr06	45447204	1-dom-ref	5.31	6.35	− 1.116	6.31	− 1.108
acb	chr06_48675074	chr06	48675074	2-dom-ref	5.45	6.35	− 1.116	6.31	− 1.108
acb	solcap_snp_c2_9011	chr06	53033201	1-dom-ref	5.31	5.34	− 0.194		
acb	chr06_56066717	chr06	56066717	2-dom-alt	5.55	6.64	− 0.863	6.7	− 0.864
acb	chr07_52271898	chr07	52271898	1-dom-ref	5.31	8.64	− 0.649	8.46	− 0.641
acb	solcap_snp_c2_42757	chr07	53172608	1-dom-alt	5.55	5.66	0.283		
acb	chr07_53228342	chr07	53228342	2-dom-alt	5.55	6.88	− 0.973	6.68	− 0.956
acb	chr08_53003386	chr08	53003386	2-dom-alt	5.55	6.09	− 1.083	6.12	− 1.083
acb	chr08_54843929	chr08	54843929	1-dom-alt	5.55	6.38	− 0.208	6.59	− 0.211
acb	solcap_snp_c2_19125	chr08	54879224	1-dom-ref	5.31	6.28	0.197	6.46	0.200
acb	chr08_55990271	chr08	55990271	general	5.57	7.21		7.33	
acb	chr08_57155618	chr08	57155618	2-dom-alt	5.55	6.09	− 1.083	6.12	− 1.083
acb	chr08_57244960	chr08	57244960	1-dom-alt	5.55	6.09	1.083	6.12	1.083
acb	solcap_snp_c2_16998	chr08	57262719	1-dom-ref	5.31	6.09	− 1.083	6.12	− 1.083
acb	chr08_57397431	chr08	57397431	2-dom-ref	5.45	6.09	− 1.083	6.12	− 1.083
acb	chr08_58958179*	chr08	58958179	1-dom-ref	5.31	6.09	− 1.083	6.12	− 1.083
acb	chr09_8432648	chr09	8432648	2-dom-ref	5.45	6.27	− 1.103	6.17	− 1.092
acb	chr09_60628740	chr09	60628740	2-dom-alt	5.55	6.06	− 0.254	6.08	− 0.255
acb	chr09_64429520	chr09	64429520	1-dom-ref	5.31	6.27	− 1.103	6.17	− 1.092
acb	chr09_67244185	chr09	67244185	2-dom-ref	5.45	6.27	− 1.103	6.17	− 1.092
acb	chr10_1154771	chr10	1154771	2-dom-alt	5.55	6.82	0.979	6.65	0.963
acb	chr10_46824811	chr10	46824811	1-dom-alt	5.55	6.9	1.167	6.82	1.157
acb	chr10_54965887	chr10	54965887	1-dom-ref	5.31	7.22	− 0.768	7.13	− 0.760
acb	chr10_56216532*	chr10	56216532	1-dom-alt	5.55	6.77	0.694	6.8	0.693
acb	chr10_57105445	chr10	57105445	additive	5.57	5.73	− 0.077	5.76	− 0.077
acb	chr12_576984	chr12	576984	2-dom-alt	5.55	6.29	− 1.110	6.21	− 1.100
acb	chr12_3279376	chr12	3279376	1-dom-alt	5.55	6.29	1.110	6.21	1.100
acb	chr12_7052755	chr12	7052755	2-dom-alt	5.55	6.29	− 1.110	6.21	− 1.100
acb	chr12_9888881	chr12	9888881	1-dom-ref	5.31	6.29	− 1.110	6.21	− 1.100
acb	chr12_48900155	chr12	48900155	2-dom-alt	5.55	6.29	− 1.110	6.21	− 1.100
atw	chr01_66991241*	chr01	66991241	additive	5.57	5.71	5.560	5.96	5.763
atw	chr02_40212654	chr02	40212654	2-dom-alt	5.55	6.11	90.115	6.11	90.111
blk	solcap_snp_c2_26938	chr08	6656143	2-dom-alt	4.78	4.94	5.170		
blk	chr08_13120400	chr08	13120400	general	5.51			6.45	
bls	chr01_1641563	chr01	1641563	2-dom-ref	5.45	9.09	12.329	8.9	12.220
bls	chr01_82281085	chr01	82281085	2-dom-alt	5.54	11.36	20.895	11.29	20.845
bls	chr01_84329619	chr01	84329619	general	5.57	7.18		7.27	
bls	chr02_28375147	chr02	28375147	1-dom-ref	5.31	5.42	14.140	5.46	14.227
bls	chr02_40968720	chr02	40968720	2-dom-ref	5.45	8.86	25.712	8.93	25.939
bls	chr02_43370405	chr02	43370405	1-dom-ref	5.31	8.86	25.712	8.93	25.939
bls	chr03_58294455	chr03	58294455	2-dom-alt	5.54	8.81	25.692	8.92	25.938
bls	chr04_61675520	chr04	61675520	1-dom-ref	5.31	5.43	14.080	5.37	14.059
bls	chr04_67414969	chr04	67414969	1-dom-ref	5.31	6.32	15.362	6.49	15.616
bls	chr04_67446650*	chr04	67446650	general	5.57			5.6	
bls	chr05_2050496	chr05	2050496	2-dom-alt	5.54	8.78	25.631	8.85	25.843
bls	chr05_3709550	chr05	3709550	1-dom-ref	5.31	8.78	25.631	8.85	25.843
bls	chr05_19376446	chr05	19376446	2-dom-alt	5.54	8.78	25.631	8.85	25.843
bls	chr05_46796271	chr05	46796271	general	5.57	8.12		8.12	
bls	chr05_52371889	chr05	52371889	2-dom-ref	5.45	7.89	− 10.688	7.66	− 10.557
bls	chr05_52371953	chr05	52371953	1-dom-alt	5.55	7.89	10.688	7.66	10.557
bls	chr07_52029903	chr07	52029903	general	5.57			5.58	
bls	chr07_52131036	chr07	52131036	2-dom-ref	5.45	5.64	14.681	5.61	14.692
bls	chr08_45397313	chr08	45397313	2-dom-alt	5.54	6	5.989	6.02	6.036
bls	chr08_49604649	chr08	49604649	1-dom-ref	5.31	8.97	25.819	9.01	25.983
bls	solcap_snp_c1_15692	chr08	49820861	1-dom-alt	5.55	8.97	− 25.819	9.01	− 25.983
bls	solcap_snp_c2_28522	chr08	50650453	diplo-general	5.57	8.73		8.74	
bls	chr08_51315335	chr08	51315335	general	5.57	8.18		8.29	
bls	chr08_53154983	chr08	53154983	2-dom-ref	5.45	6.03	9.428	6	9.539
bls	chr09_1542138	chr09	1542138	1-dom-alt	5.55	5.62	− 8.480	6.03	− 8.907
bls	chr09_29876333	chr09	29876333	general	5.57	7.3		7.29	
bls	chr10_6048550	chr10	6048550	general	5.57	6.79		6.84	
bls	chr10_44024724	chr10	44024724	2-dom-ref	5.45	6.74	− 15.808	6.81	− 15.941
bls	chr11_32771986	chr11	32771986	general	5.57	7.09		7.22	
brt	solcap_snp_c2_11584	chr02	6094025	additive	5.57			5.69	− 0.425
brt	chr02_28929643	chr02	28929643	2-dom-alt	5.55	5.76	− 2.214	5.62	− 2.190
brt	chr02_31549993	chr02	31549993	2-dom-ref	5.45	7.38	− 3.450	7.19	− 3.404
brt	solcap_snp_c2_15066	chr02	43229112	2-dom-alt	5.55	6.08	3.073	6.03	3.059
brt	chr02_43238350	chr02	43238350	2-dom-ref	5.45	6.08	− 3.073	6.03	− 3.059
brt	chr06_38005283	chr06	38005283	additive	5.57	5.84	− 0.288	5.86	− 0.289
csc	solcap_snp_c2_41335	chr01	64463239	2-dom-alt	5.55	6.17	− 7.433	5.7	− 7.116
csc	solcap_snp_c2_41336	chr01	64463260	2-dom-ref	5.45	6.17	7.433	5.7	7.116
csc	chr08_34540716	chr08	34540716	1-dom-ref	5.31	5.33	6.216		
csc	chr11_40241089	chr11	40241089	2-dom-alt	5.55	5.86	16.194	5.71	15.776
csc	chr12_11240374	chr12	11240374	2-dom-ref	5.45	7.13	13.213	6.9	12.828
csc	chr12_12881745	chr12	12881745	2-dom-ref	5.45	5.51	13.818		
drm	chr10_58099346	chr10	58099346	1-dom-alt	5.55	7.99	− 2.884	7.76	− 2.848
drm	chr12_57450585	chr12	57450585	2-dom-alt	5.55			5.64	3.573
eye	chr03_60298153	chr03	60298153	additive	5.57	5.79	− 0.644		
eye	chr04_5763134	chr04	5763134	2-dom-alt	5.55	6.23	− 1.572	5.75	− 1.509
eye	chr04_10456693	chr04	10456693	2-dom-ref	5.45	7.38	− 1.687	7.19	− 1.661
eye	chr04_46696845	chr04	46696845	2-dom-alt	5.55	8.87	− 3.072	8.46	− 2.997
eye	chr06_40734919	chr06	40734919	1-dom-alt	5.55	5.76	− 0.591	6.16	− 0.617
eye	chr06_40735030	chr06	40735030	additive	5.57	6.31	− 0.534	6.73	− 0.556
eye	chr07_53711618	chr07	53711618	2-dom-alt	5.55	7.21	− 1.268	6.67	− 1.216
eye	chr07_53926257	chr07	53926257	2-dom-ref	5.45	8.89	− 3.098	8.36	− 2.990
eye	solcap_snp_c2_45881	chr09	53729760	2-dom-ref	5.45	13.22	− 2.720	12.73	− 2.663
eye	solcap_snp_c2_25485	chr10	49589876	general	5.57	15.78		16.15	
eye	solcap_snp_c2_57641	chr10	50246851	1-dom-alt	5.55	15.01	− 0.667	15.23	− 0.671
eye	chr10_50383861	chr10	50383861	additive	5.57	14.81	0.357	15.08	0.360
eye	chr11_3404728	chr11	3404728	2-dom-alt	5.55	10.8	− 2.024	10.29	− 1.965
eye	chr11_6166082	chr11	6166082	2-dom-alt	5.55	5.57	− 0.450		
eye	chr11_7400337	chr11	7400337	2-dom-ref	5.45	6.81	0.951	6.4	0.915
eye	chr11_10058756	chr11	10058756	2-dom-alt	5.55	6.08	− 1.338	6.02	− 1.321
eye	chr12_1449252	chr12	1449252	2-dom-alt	5.55	13.05	− 2.690	12.62	− 2.644
fsh	chr02_32301311	chr02	32301311	1-dom-ref	5.31	6.97	3.247	8.28	3.340
fsh	chr02_45195794	chr02	45195794	2-dom-ref	5.45			5.64	1.655
fsh	chr03_41501750	chr03	41501750	diplo-general	5.57	10.58			
fsh	chr03_41527317*	chr03	41527317	additive	5.57	9.59	0.469		
fsh	solcap_snp_c2_46603	chr03	42455195	2-dom-alt	5.55	7.63	− 0.555	6.04	− 0.445
fsh	solcap_snp_c2_20227	chr03	43546430	1-dom-alt	5.55	22.36	1.107	15.86	0.906
fsh	chr03_44031918	chr03	44031918	2-dom-ref	5.45	10.1	− 0.705		
fsh	chr03_44033492	chr03	44033492	1-dom-ref	5.31	31.35	− 1.296	24.77	− 1.128
fsh	chr03_45444220	chr03	45444220	2-dom-alt	5.55	6.12	0.529		
fsh	chr03_45449131	chr03	45449131	1-dom-alt	5.55	25.27	1.112	18.47	0.928
fsh	solcap_snp_c2_25653	chr03	47133164	2-dom-alt	5.55	13.15	0.761	8.73	0.585
fsh	chr03_47342606	chr03	47342606	1-dom-ref	5.31	25.76	− 1.170	18.6	− 0.975
fsh	chr03_48304511	chr03	48304511	2-dom-ref	5.45	17.42	− 0.916	10.82	− 0.697
fsh	chr03_50343327	chr03	50343327	1-dom-alt	5.55	11.55	0.703	7.05	0.507
fsh	solcap_snp_c1_398	chr03	50462478	2-dom-alt	5.55	5.63	0.426		
fsh	chr03_50472590	chr03	50472590	2-dom-ref	5.45			6.52	− 0.448
fsh	chr05_10586111	chr05	10586111	1-dom-alt	5.55			6.09	− 2.098
fsh	chr07_53228342	chr07	53228342	2-dom-alt	5.55	7.23	3.352	7.99	3.302
fsh	chr07_55218233	chr07	55218233	1-dom-alt	5.55	5.95	− 2.071	6.2	− 1.979
fsh	chr08_4816673	chr08	4816673	2-dom-alt	5.55			5.79	1.826
fsh	chr10_1154771	chr10	1154771	2-dom-alt	5.55	7.18	− 3.345	8.08	− 3.332
fsh	chr10_53541706	chr10	53541706	1-dom-alt	5.55			5.81	− 2.037
fsh	chr11_44481634	chr11	44481634	2-dom-ref	5.45			5.93	1.791
hbr	chr03_47183592	chr03	47183592	general	5.57	5.79		5.83	
hbr	solcap_snp_c2_50302	chr05	4944682	additive	5.57	6.57	0.031	6.47	0.031
hbr	chr10_4673749	chr10	4673749	1-dom-alt	5.55	5.73	0.100	5.83	0.101
hbr	chr12_4567466	chr12	4567466	general	5.57	5.8			
shp	solcap_snp_c2_25471	chr10	49660365	1-dom-alt	5.55	13.98	− 0.819	13.84	− 0.818
shp	solcap_snp_c1_8019	chr10	49714737	2-dom-alt	5.55	11.09	− 0.687	11.08	− 0.689
shp	chr10_50383861	chr10	50383861	additive	5.57	20.52	0.545	20.4	0.545
tps	solcap_snp_c2_54598	chr05	13391108	2-dom-alt	5.55	6.04	− 0.737	6.07	− 0.744
tps	chr06_51374524	chr06	51374524	2-dom-ref	5.45	6.62	0.641	6.52	0.642
tps	chr06_56546361	chr06	56546361	additive	5.57	5.72	0.199		
ttu	chr01_66016265	chr01	66016265	additive	5.57	5.64	22.285	5.58	22.371
ttu	chr04_63755427	chr04	63755427	general	5.57	6.12		6.04	
ttu	chr09_50878917	chr09	50878917	1-dom-alt	5.55	5.91	43.026	5.74	42.758
uni	solcap_snp_c2_25510	chr10	49532389	2-dom-ref	5.45	5.77	0.546	5.7	0.547
spr	chr01_6237003	chr01	6237003	general	5.57	5.73		6.3	
spr	chr01_15064177*	chr01	15064177	general	5.57	6.47		6.97	
spr	solcap_snp_c2_35518	chr01	62156035	general	5.57	5.8		6.21	
spr	chr01_63714469	chr01	63714469	general	5.57	6.67		7.15	
spr	chr01_65999314	chr01	65999314	diplo-additive	5.57	5.64	5.669		
spr	solcap_snp_c2_16348	chr02	13859826	general	5.57	5.8		6.11	
spr	solcap_snp_c2_51115	chr02	28245319	general	5.57			5.72	
spr	chr03_34558316	chr03	34558316	general	5.57	5.86		6.1	
spr	solcap_snp_c2_48371	chr03	38808607	general	5.57	5.9		6.12	
spr	solcap_snp_c1_7112	chr03	53165918	general	5.57	5.63		5.79	
spr	chr04_61731724	chr04	61731724	general	5.57	6.24		6.52	
spr	solcap_snp_c2_50532	chr05	32496724	general	5.57	5.92		6.32	
spr	solcap_snp_c2_57242	chr05	36587438	general	5.57	5.82		6.15	
spr	chr05_46517291	chr05	46517291	general	5.57	5.82		6.04	
spr	chr05_52063877	chr05	52063877	2-dom-alt	5.55	7.1	52.604	7.39	54.032
spr	solcap_snp_c2_27564	chr06	3758998	general	5.57	5.62		5.98	
spr	chr06_59041055	chr06	59041055	general	5.57	6.21		6.31	
spr	chr07_332930	chr07	332930	2-dom-alt	5.55	6.92	52.320	7.22	53.709
spr	solcap_snp_c2_6601	chr07	10075616	general	5.57			5.72	
spr	solcap_snp_c2_56547	chr07	17169117	general	5.57	5.85		6.21	
spr	solcap_snp_c2_34561	chr07	21038902	general	5.57	5.66		6.02	
spr	solcap_snp_c1_16450	chr07	25252634	general	5.57	5.62		5.89	
spr	solcap_snp_c2_31366	chr07	30807906	general	5.57	5.62		5.89	
spr	solcap_snp_c2_1993	chr07	38542005	general	5.57	5.75		6.05	
spr	chr07_44828209	chr07	44828209	2-dom-alt	5.55	5.99	26.058	6.11	26.373
spr	chr07_45262541	chr07	45262541	general	5.57	6.04		6.08	
spr	solcap_snp_c2_38764	chr07	49065413	general	5.57	5.69		5.84	
spr	solcap_snp_c2_28171	chr07	52491515	general	5.57	5.65		5.74	
spr	chr09_10090278	chr09	10090278	general	5.57	5.77		5.74	
spr	chr11_644550	chr11	644550	additive	5.57	5.79	7.695		
spr	chr11_1115236	chr11	1115236	1-dom-alt	5.55	6.73	10.448	6.18	10.060
spr	chr11_2824581	chr11	2824581	2-dom-alt	5.55	7.1	53.389	7.43	54.765
spr	chr11_10344556	chr11	10344556	2-dom-ref	5.45	7.1	53.389	7.43	54.765
spr	chr11_45525259	chr11	45525259	1-dom-alt	5.55	6.49	7.404	6.39	7.341
spr	solcap_snp_c2_34194	chr11	46392768	general	5.57	5.85		6.1	
spr	chr12_56280690	chr12	56280690	general	5.57	5.83		6.06	
yld	chr01_48616298	chr01	48616298	2-dom-ref	5.45			5.51	− 3.622
yld	chr01_78786092	chr01	78786092	1-dom-ref	5.31	5.37	− 16.457	5.46	− 16.492
yld	chr01_78786162	chr01	78786162	2-dom-ref	5.45			5.46	− 16.492
yld	chr02_25482863	chr02	25482863	2-dom-ref	5.45			5.48	− 5.077
yld	chr03_35821952	chr03	35821952	additive	5.57	5.92	2.596	6.64	2.801
yld	chr03_46407327	chr03	46407327	1-dom-ref	5.31			5.44	− 11.171
yld	chr04_9446052	chr04	9446052	2-dom-ref	5.45	5.64	2.269		
yld	chr06_56005795	chr06	56005795	diplo-additive	5.57	6.65	2.820	6.37	2.746
yld	chr08_57420725	chr08	57420725	general	5.57	6.37		6.25	
yld	chr12_16077270	chr12	16077270	2-dom-alt	5.55	6.84	− 12.819	7	− 12.904

^a^for six MTAs (marked with asterisk), the highest significance was observed with the diploidized model, but the details presented are from the next best tetraploid model

## Discussion

### GBS targets genic regions and provides effective assessment of population structure and LD decay rates

A number of restriction-enzyme pairs were evaluated for constructing potato GBS libraries where the *Pst*I–*Mse*I pair was found to be most effective in terms of the number and distribution of GBS-tags in the optimum range for constructing GBS libraries. Furthermore, visualization of the genome-wide distribution of the SNPs obtained from *Pst*I–*Mse*I GBS libraries revealed enrichment of GBS SNPs in the euchromatic regions and their avoidance in repetitive regions with the SNP distribution closely matching that of gene density (Fig. 2). The reported GBS method uses a methylation-sensitive restriction enzyme *Pst*I as the ‘rare’ cutter, which avoids repetitive regions of the genome. Consequently, despite it being a non-targeted approach GBS-tags are mostly confined to the euchromatic fraction of the genome. Further genomic annotation using SNPeff (Cingolani et al. 2012) revealed 10.4% of GBS SNPs are located in intergenic regions, while 89.6% in genic (47.6%) or gene-associated (42%; 5 k bases up/downstream of gene boundaries) parts of the genome.

In accordance with the previous genetic studies (Dhoop et al. 2008; Sharma et al. 2018; Stich et al. 2013) citing a weak population structure in potato, although there was no clear visual separation of clones into distinct subpopulations, the genetic characterization of the potato panel using GBS and Infinium SNPs detected subtle grouping of the clones into three subpopulations. Moreover, DAPC single-axis density plot revealed enhanced level of discrimination among the three subpopulation groups by using GBS SNPs than that achieved by the Infinium SNPs. Both SNP sets were also utilized to assess LD rates in the potato panel and the LD decay estimates obtained here were found to be comparable to those previously reported (Dhoop et al. 2010; Sharma et al. 2018; Simko et al. 2006; Vos et al. 2017) and contrast the only study (Stich et al. 2013) reporting very sharp LD decay in potato. As compared to the rest of the chromosomes, chromosome 8 has been reported to display extremely conserved levels of LD_1/10,90_, for example, 8 Mb (Berdugo-Cely et al. 2017) and 20.04 Mb (Sharma et al. 2018). While the current study reports a similar trend of LD decay for chromosome 8 (LD_1/10,90_: 25.23 Mb) using Infinium array SNPs, the LD_1/10,90_ measure for chromosome 8 using GBS SNPs is only 1.48 Mb which aligns well with the LD decay rates observed for the other chromosomes. The enhanced efficiency of GBS-SNPs in capturing LD decay rates could be attributed to the increased enrichment of GBS SNPs in the euchromatic region as well as more effective coverage of the heterochromatic parts of the genome as compared to Infinium SNPs (Fig. 3). Although the pattern (Fig. 3) and proportion (data not shown) of GBS SNPs in euchromatic and heterochromatic regions, as described by Sharma et al. (2013) and Leyva-Perez et al. (2022), for chromosome 8 did not significantly differ from rest of the chromosomes, the pronounced shift in LD decay rate for this chromosome using GBS SNPs is somewhat intriguing.

### Complex interplay of traits in the potato panel

The association panel was phenotyped for sixteen production, agronomic and processing traits. Significant correlations were observed between some traits while correlations between most trait-pair combinations were non-significant (Fig. 8). For example, average tuber weight shows a strong negative correlation (− 0.68) with total tubers per plant, but a strong positive correlation (+ 0.63) with tuber yield. These observations are expected as larger tubers result in a greater yield, and fewer tubers per plant would result in less competition between tubers for photosynthates leading to an increase in tuber size. Tuber dry matter shows significant negative correlations with yield (− 0.23) and average tuber weight (− 0.34) which aligns well with the tendency for greater yields if tubers contain more water. Tubers per plant and tubers per stem show a positive strong correlation (+ 0.32) as expected. Tuber dry matter also shows a significant negative correlation with tuber skin brightness (− 0.33) which potentially could be an artefact of selection as varieties bred for fresh market need a better skin finish to gain acceptance but for the processing sector, largely favouring varieties with higher dry matter, skin finish is far less important. A negative correlation (− 0.27) between tuber dry matter and tuber eye depth (EYE scoring: 1 deep, 9 shallow) is interesting and emphasizes the challenges for breeding varieties suitable for processing (i.e. with high dry matter content) while selecting for shallow eye depth. Tuber eye depth also shows a strong positive correlation with tuber shape (0.47; SHP: 1–6, 1 round), which in turn displays a significant negative correlation with tuber dry matter (− 0.24). The latter could also be affected by selection as higher dry matter and round tuber shape are amongst the highly desirable traits for the processing industry, mainly the potato crisp/chip market segment. Tuber shape shows a significant correlation (− 0.42) with uniformity (UNI: 1–9, 9 very uniform). This potentially could be due to the observations that round tubers are more likely to retain uniformity in shape as they increase in size, however, the longer tubers will be more prone to display variation in tuber length because of the possibility of the presence of a number of smaller tubers which haven’t yet fully elongated. After cooking blackening displays significant correlations with average tuber weight (0.22) and tuber yield (0.30) but a strong negative correlation with tuber dry matter (− 0.42; ACB scoring, 1 severe). Significant correlation between tuber bruising (enzymatic browning) and dry matter is previously reported (Urbany et al. 2011) and the ACB trait (non-enzymatic browning) assessed here seems to display similar patterns. These observations also align well with the negative correlation of tuber dry matter described above with tuber yield and average tuber weight. After cooking blackening also shows a significant negative correlation (− 0.26) with tuber flesh colour suggesting white tubers (low FSH value) have on average a lower tendency to darken after cooking (high ACB value).

### GBS enhances marker saturation and QTL resolution for GWAS

GWAS were performed for all sixteen traits assessed in the study using a combined marker set of GBS and Infinium SNPs. GWAS using individual SNP datasets (viz. GBS and Infinium) were also performed but results mainly using the combined SNP dataset are discussed here. As previously reported by Sharma et al. (2018), the performance of *K* and *QK* models was the most effective and generally in agreement with each other while the *N* model was least efficient amongst the four tested models in controlling population structure effects (Supplementary Table S8, Supplementary Fig. S5). For almost every trait, the QTL–MTA observed in *K* and *QK* models corroborated each other. However, in a few cases the results differed (i.e. QTL–MTA absent in either *K* or *QK* model) for one (tubers per stem, height breadth ratio, tuber skin brightness and tuber dry matter), two (common scab, after cooking blackening, black dot, black scurf and eye depth), four (tuber sprouting), five (tuber yield) and eleven (tuber flesh colour) QTL-MTAs. Most traits had at least a single QTL–MTA obtained from both marker sets, while QTL-MTAs for one trait (tuber uniformity) belonged to Infinium array and for four traits (average tuber weight, tuber dry matter, total tubers per plant and tuber yield) to GBS SNPs only. For ‘black dot’, GBS–Infinium–GWAS did not result in any MTAs possibly due to the increased significance threshold with the higher number of markers employed, and results from the GBS–GWAS and Infinium–GWAS are included. Although the described QTL-MTAs for ‘black dot’ (obtained using individual SNP dataset GWAS) did not achieve the set significance threshold using the combined SNP set analysis, they still showed the highest association strength in the reported genetic model categories for GBS–Infinium–GWAS. For the non-redundant set of 189 QTL-MTAs obtained from GBS–Infinium–GWAS, the majority of significant QTL-MTAs obtained were from GBS SNPs (42 Infinium vs. 147 GBS QTL-MTAs) highlighting their proximity to the causal variation and increased marker saturation and resolution achieved through GBS. The GWAS results for each of the traits studied are discussed in the light of previous published studies in more detail below:

#### After cooking blackening

After-cooking blackening (ACB) or darkening results from a non-enzymatic grey–black discoloration of the potato tubers after they are cooked using methods such as boiling, baking, frying and dehydration (Hughes and Swain 1962). ACB has a direct impact on the quality and marketability of potato products and is regarded as one of the most undesirable quality defects of potatoes. Previously, QTL for ACB have been reported on chromosomes 2, 4, 6, and 11 including an undefined homology group ‘C’ (Bradshaw et al. 2008) in a tetraploid biparental mapping population. However, the current GWAS for ACB revealed QTL-MTAs on all chromosomes except 4 and 11 (Table 5; Figs. 9, 10; Supplementary Fig. S6). The very poor resolution of the genetic map in the previous study, and also the lack of sequence-tagged markers prohibit detailed comparisons of the common QTL (chromosomes 2 and 6) identified in the two studies. However, the common QTL reported here appear to be in approximately the same locations as those detected in the earlier publication, that is towards the ‘bottom’ end of chromosome 2 and in the ‘mid-to-bottom’ region of chromosome 6.

Besides ACB, potato also exhibits other forms of tuber darkening, such as tuber bruising, resulting from enzymatic reactions mainly controlled by polyphenol oxidases (PPOs). Chi et al. (2014). A previous study (Urbany et al. 2011) has reported tuber bruising QTL on chromosome 8 without providing precise genomic location details, and a recent study by Angelin-Bonnet et al. (2023) also detected QTL for this trait on chromosomes 8 (~ 46 Mb, DMv4.03 (Sharma et al. 2013)) in close vicinity to the genomic location of tuber specific StPPOs (45.8 Mb; Chi et al. 2014) catalysing the production of melanin pigments that gives the bruising colour. Both studies also reported additional QTLs on chromosomes 2 and 3 (Urbany et al. 2011), and 1 and 7 (Angelin-Bonnet et al. 2023). The interval of chromosome 8 QTLs for ACB detected here is also located in the same region as tuber specific PPOs possibly with revised genomic coordinates (53–59 Mb) in the latest DM assembly version 6.1 (Pham et al. 2020) and in close vicinity to a PPO domain containing protein (48.2 Mb, DMv6.1). Moreover, one of the QTLs on chromosome 3 identified here is located close to the putatively annotated genes (4-coumarate:CoA ligase at 45.7 Mb and Glycogen/starch synthases, ADP-glucose type at 47.1 Mb) reported to be implicated in tuber bruising by Urbany et al. (2011). These observations reveal the cross-talk between pathways controlling both enzymatic and non-enzymatic discoloration of potato tubers and provide effective breeding targets for developing potatoes resistant to tuber darkening caused by multiple factors.

#### Black dot

Black dot, caused by *Colletotrichum coccodes*, was initially considered as a mild disease of potato, largely infecting weakened plants. However, in the last two decades the pathogen (*C. coccodes*) has been reported to infect roots and stems quite early in the growing season, causing early foliage death either by itself or in association with other pathogens, reducing plant and root growth as well as leading to reduced potato yields (Johnson et al. 2018). Furthermore, at the tuber phase the fungus infection causes unsightly potato blemishing, affecting tuber quality and marketability including significant water losses during storage, a factor receiving increased economic impact due to the changes in the marketing of fresh tubers, particularly with respect to the growing demand for washed potatoes (Massana-Codina et al. 2021). The GWAS for black dot performed here identified QTL-MTAs on chromosome 8 (Table 5; Figs. 9, 10; Supplementary Fig. S6). To the best of our knowledge, there have been no previous studies mapping this trait in potato, and the findings reported here could pave the way for gaining a basic understanding of genetic resistance to black dot with a view to developing black dot resistant cultivars.

#### Black scurf

Black scurf, caused by the basidiomycete *Thanatephorus cucumeris (Rhizoctonia solani)*, refers to the formation of sclerotia on potato tubers (Carling et al. 1989). The disease causes substantial yield reductions ranging from 5 to 10% including economic losses due to significant decreases in quality especially in washed tubers with fine skin (Errampalli and Johnston 2001). To date, no genetic study has been conducted to identify genetic factors controlling black scurf in potato. The black scurf GWAS reported here detected significant QTL-MTAs on chromosomes 1, 2, 3, 4, 5, 7, 8, 9, 10 and 11 (Table 5; Figs. 9, 10; Supplementary Fig. S6), suggesting a very complex genetic architecture of susceptibility to this economically important disease.

#### Common scab

Common scab, caused by Streptomyces species such as *Streptomyces scabies*, *S. acidiscabies* and *S. turgidiscabies* (Lambert and Loria 1989; Miyajima et al. 1998), is an unsightly blemish disease of potatoes occurring worldwide. Previous genetic mapping studies have identified common scab QTLs on several chromosomes viz., 1, 2, 3, 4, 6, 9, 11 and 12 (Bradshaw et al. 2008; Braun et al. 2017; Enciso-Rodriguez et al. 2018; Kaiser et al. 2020; Koizumi et al. 2021; Pereira et al. 2021a; Yuan et al. 2020) using diploid and tetraploid populations and deploying a range of marker types. GWAS here identified QTL-MTAs on chromosomes 1, 8, 11 and 12 (Table 5; Figs. 9, 10; Supplementary Fig. S6). Koizumi et al. (2021) report no agreement in regard to QTL locations and their genetic modes among the results of all previous investigations including that from their own study. However, apart from a chromosome 8 QTL, all other QTL observed here find good correspondence with the previous studies. The chromosome 1 QTL–MTA at ~ 64.5 Mb is in close vicinity to the QTL region (73.7 Mb to 75.3 Mb) on the same chromosome detected by Kaiser et al. (2020). Koizumi et al. (2021) also recognized effective SNPs in this region, but the significance level was below the set threshold possibly due to the small population size of their GWAS panel as postulated by them. The chromosome 11 QTL–MTA at ~ 40.2 Mb (chr11_40241089) reported here corroborates the chromosome 11 QTLs (35 cM and 41.2 cM) described by Braun et al. (2017) as inferred (1 cM = ~ 0.9 Mb) by equating the total genetic map length (822 cM) the latter study obtained to the physical size (741.6 Mb) of the published potato genome (Pham et al. 2020). Similarly, the chromosome 12 QTL–MTA at ~ 11.2 Mb (chr12_11240374) detected here is in close proximity to the chromosome 12 MTAs (at ~ 9.5 Mb) reported by Yuan et al. (2020) with both studies reporting ‘duplex dominance’ gene action model for their respective MTAs. This is in disagreement to the observations made by Koizumi et al. (2021) that most ‘common scab’ QTLs reported in different studies don’t correspond with each other. Furthermore, the validation of three QTL regions (chromosomes 1, 11 and 12) observed here with the previously described ones**,** suggests the robustness and higher transferability of these MTAs to a wider germplasm. The chromosome 8 QTL–MTA reported is novel to the current study and provides an additional breeding target to develop common scab resistant potato varieties.

#### Tuber shape uniformity

Tuber shape uniformity is an important trait in potato breeding which has significant impact on consumer acceptance and industrial processing. Studies (Bradshaw et al. 2008; Hara-Skrzypiec et al. 2018; Śliwka et al. 2008) have been conducted to unravel the genetic factors controlling this trait, with QTLs identified on several chromosomes (1, 2, 3, 4, 5, 8, and 11). Previously, this trait has been assessed in biparental mapping populations using the term ‘regularity of tuber shape’ (here after referred to as ‘regularity’) which includes many components such as depths of indentations, presence of various tuber defects and all other deviations from an ‘ideal’ tuber shape (Domański 2001). However, not all studies fully define the metrics used to score the regularity trait. There are no studies reported on tuber uniformity per se, however, as the regularity trait has the tuber shape uniformity as one of the major components, the results are discussed in the context of the previous studies. Hara-Skrzypiec et al. (2018) reported seven QTL for regularity trait on chromosomes 1, 3, 4, 5, and 8. On some of these previously reported chromosomes (1, 4 and 8) and also chromosome 12, the results obtained here revealed QTL peaks reaching close to the significance level, but the set stringency threshold was not achieved (Figs. 9, 10; Supplementary Fig. S6). GWAS here also detected significant tuber uniformity QTL localized in *Ro* locus on chromosome 10 (solcap_snp_c2_25510, chr10:49,532,389 bp, Table 5) which, to the best of our knowledge, is not reported previously.

#### Tuber skin brightness (texture)

Tuber skin texture is an important trait in potato because a smooth ‘skin finish’ is highly desirable for fresh-market varieties. The only published study (McCord et al. 2011) mapping this trait in potato detected multiple QTL (19 in total) spanning chromosomes 2–6, 9, 10 and 12. GWAS here identified significant QTL on chromosomes 2 and 6 (Table 5; Figs. 9, 10; Supplementary Fig. S6). Two of the five QTL-MTAs (at ~ 43.2 Mb, Table 5) on chromosome 2 detected here are in close vicinity to one of the three chromosome 2 QTLs identified by McCord et al. (2011) mapping to 70 cM (~ 49 Mb). The genetic to physical distance conversion is calculated as described above (‘Atlantic’ parent; total genetic map length: 1059.4 cM; cM-to-Mb conversion factor: 0.7; physical genome size: 741.6 Mb (Pham et al. 2020)). The chromosome 6 QTL–MTA and the remaining chromosome 2 QTL-MTAs were at different genomic locations than reported in McCord et al. (2011) and are novel to this study. GWAS here also revealed effective QTL peaks on chromosomes 1, 3, 4, 9 and 12 but remained marginally below the set significance threshold (Figs. 9, 10; Supplementary Fig. S6). Of these, QTL on chromosomes 3 and 12 show a good correspondence with those reported by McCord et al. (2011), while chromosome 1 QTL is absent, chromosome 4 QTL has only one marker (without any location details) and the chromosome 9 QTL is in a discordant location (than observed here) in the work presented by these authors.

#### Tuber flesh colour

Tuber flesh colour in potato is determined primarily by the carotenoid content, and its intensity varies from white to deep yellow, in the absence of other types of pigmented compound (e.g. anthocyanins). The yellow flesh locus (*Y*-locus) controlling the white-to-yellow flesh colour in potato maps to chromosome 3 (Bonierbale et al. 1988; Jacobs et al. 1995). The presence of a dominant allele ‘*Or’* at *Y*-locus or in close vicinity determines orange flesh colour (Brown et al. 1993). GWAS conducted here revealed QTL on chromosomes 2, 3, 5, 7, 8, 10 and 11 (Table 5; Figs. 9, 10; Supplementary Fig. S6). The strongest association (chr03_44033492) was observed at 44.03 Mb on Chromosome 3, just 1.14 Mb away from β-carotene hydroxylase gene (Soltu.DM.03G018410, 42893526–42895894, DMv6.1 (Pham et al. 2020)) which is considered as the main regulator of flesh colour in potato (Kloosterman et al. 2010) and has been verified in a number of studies (D’hoop et al. 2008; Endelman and Jansky 2016; Hara-Skrzypiec et al. 2018). Besides the chromosome 3 locus, control of flesh colour by QTLs residing on several other chromosomes (1, 2, 4, 6, 7, 9, 10, 12) is also reported (Campbell et al. 2014; D’hoop et al. 2008; Endelman and Jansky 2016; Hara-Skrzypiec et al. 2018; Li et al. 2021; Śliwka et al. 2008). Our analysis revealed two QTL-MTAs (chr02_32301311 and chr02_45195794) on chromosome 2 at 32.3 Mb and 45.2 Mb. The latter QTL–MTA (at 45.2 Mb) shows a good agreement with one of the QTL (chromosome 2, 48.52 Mb, DMv4.03 (Sharma et al. 2013)) for anthocyanidins reported by Parra-Galindo et al. (2019), and grossly corresponds with the QTL region on the bottom part of chromosome 2 (76.2–77.5 cM; 58.1–59.1 Mb; see above for cM-to-Mb conversion) reported by Hara-Skrzypiec et al. (2018). Zhang et al. (2009), using non-sequence-tagged markers, identified QTL for tuber flesh colour on the top, top and mid-to-bottom parts of chromosomes 5, 8, and 9, respectively. While GWAS here did not reveal QTL on chromosome 9, the QTL-MTAs detected on chromosomes 5 (chr05_10586111) and 8 (chr08_4816673) appear to be coinciding with the QTL detected by Zhang et al. (2009), albeit after accounting for possible reverse genetic orientation for chromosome 8 reported in their study. D’hoop et al. (2008) reported a tuber flesh colour QTL on chromosome 7 which, though positional details are not divulged, supports the two QTL regions (chr07_53228342 and chr07_55218233) on chromosome 7 identified here. Li et al. (2021) found a major QTL for tuber flesh colour on chromosome 10 co-localizing with the *I* gene (*StMYB88*) reported to control tuber peel colour. These authors further revealed that *StMYB88* and one other Myb family gene (*StMYB89*) located on chromosome 10 (51.7–52.3 Mb, DMv4.03) were closely related to regulating anthocyanin biosynthesis of tuber flesh. Parra-Galindo et al. (2019) also detected several QTL (10 QTL at ~ 52 Mb and 1 QTL at ~ 57.3 Mb, DMv4.03 (Sharma et al. 2013)) for anthocyanidins content on chromosome 10 in the similar region. Furthermore, a QTL for ‘Pigmented skin intensity’ (*Pf*) on chromosome 10 (53.48–56.11 Mb, DMv4.03 (Sharma et al. 2013)) is reported by Endelman and Jansky (2016) supporting the genetic interactions controlling tuber flesh and skin colour stated by Li et al. (2021) as described above. GWAS here identified two QTL-MTAs on chromosome 10 (chr10_1154771 and chr10_53541706). Of these, the latter QTL–MTA at ~ 53.5 Mb coincides with the chromosome 10 QTL reported in above studies further supporting the importance of this region in regulating tuber flesh colour. The current study also detected a QTL–MTA on chromosome 11 (chr11_44481634) which finds good correspondence with a QTL (~ 40 Mb, DMv4.03 (Sharma et al. 2013)) for anthocyanidins content reported by Parra-Galindo et al. (2019) on the same chromosome.

#### Tuber sprouting (dormancy)

Tuber sprouting is one of the commercially important potato traits and critical for the long-term tuber storage required to ensure year-round availability as premature dormancy release and sprout growth in tubers during storage can result in a significant deterioration in product quality. Therefore, breeding potato cultivars with extended or otherwise modified dormancy or sprout vigour is a desirable goal (Alamar et al. 2017; Sharma et al. 2021). Tuber dormancy release and sprout growth are two different physiological phenomena and are often confounded in QTL studies, thereby contributing to the complex genetic architecture of this compound trait. Previous genetic studies (Freyre et al. 1994; Sharma et al. 2021; Simko et al. 1997; Simmonds 1964; van den Berg et al. 1996) on tuber dormancy have detected QTL effects on all chromosomes except chromosome 12. The analysis conducted here also revealed the presence of QTL on several (1, 2, 3, 4, 5, 6, 7, 9, 11 and 12) chromosomes (Table 5; Figs. 9, 10; Supplementary Fig. S6). Due to the very poor resolution of the genetic map in the previous studies, except by Sharma et al. (2021), and largely the deployment of non-sequence-tagged markers, detailed comparisons with the current study are not possible, however, many QTL reported here appear to be in approximately the same genomic locations as reported previously including the detection of novel QTL regions (Table 5). A much more recent study (Sharma et al. 2021) conducted with high map resolution reports tuber dormancy/sprouting QTL on chromosomes 1–8 and 10. Of these, chromosomes 8 and 10 QTL were not detected in the current study but all other QTL (i.e. on chromosomes 1–7) find higher correspondence with the GWAS QTL-MTAs reported here. None of the previous studies report QTL for this trait on chromosome 12 except Sharma et al. (2021) who suggest the presence of an effective QTL peak at the bottom end of this chromosome but the required statistical significance in their study was not achieved. The chromosome 12 QTL–MTA (Table 5) observed here strongly overlaps, and further strengthens, the chromosome 12 candidate QTL suggested by Sharma et al. (2021). Taken together QTL locations from current and previous studies reveal the involvement of all 12 potato chromosomes indicating a very complex genetic architecture for tuber dormancy/sprouting and that the trait is under the control of many genes acting at different loci.

#### Tuber shape

Tuber shape is one of the most important quality traits in potato for both processing industry and fresh market use. The single major QTL (*Ro* locus) controlling this trait is mapped on chromosome 10 (Endelman and Jansky 2016; Fan et al. 2022; Hara-Skrzypiec et al. 2018; Lindqvist-Kreuze et al. 2015; Pandey et al. 2022; Prashar et al. 2014; Sharma et al. 2018; Śliwka et al. 2008; van Eck et al. 1994), however, tuber shape QTLs on different chromosomes have also been detected (D’hoop et al. 2014; Hara-Skrzypiec et al. 2018; Huang et al. 2022; Lindqvist-Kreuze et al. 2015; Manrique-Carpintero et al. 2018; Park et al. 2021; Prashar et al. 2014). Tuber shape GWAS conducted here detected significant QTL–MTA (chr10_50383861; Table 5; Figs. 9, 10; Supplementary Fig. S6) on chromosome 10 corroborating the findings described above. Significantly, there are reports that tuber shape is very largely controlled by the *StOFP20* gene on chromosome 10 (Ai et al. 2023; van Eck et al. 2022). van Eck et al. (2022) clearly demonstrate that use of RNAi ‘knock downs’ or overexpression of *StOFP20* in cultivars with round or elongated tubers can significantly alter the shape of tubers towards the opposite extreme, suggesting that the *StOFP20* gene is indeed responsible for controlling this trait in potato.

#### Tuber eye depth

Tuber eye depth is an important agronomic trait that affects the appearance and processing quality of tubers where potatoes with deep eyes contribute to significant peeling losses and are also not favoured by the consumers. To aid breeding potatoes with shallow eyes and uniform shape, several studies have been conducted in the past to identify genetic factors underlying this trait and identified a major locus controlling eye depth on chromosome 10 including minor effect QTLs on chromosomes 1, 2, 3, 4, 5, 6, 11, and 12 (Hara-Skrzypiec et al. 2018; Li et al. 2005; Lindqvist-Kreuze et al. 2015; Pandey et al. 2022; Prashar et al. 2014; Rosyara et al. 2016; Sharma et al. 2018; Śliwka et al. 2008; Totsky et al. 2020; Zhang et al. 2022; Zhao et al. 2023). GWAS here detected the most significant QTL for tuber eye depth on chromosome 10 corroborating the findings from the previous studies, and additional significant QTL-MTAs were revealed on chromosomes 3, 4, 6, 7, 9, 10, 11 and 12 (Table 5; Figs. 9, 10; Supplementary Fig. S6). The chromosome 3 eye depth QTL reported by Prashar et al. (2014) is positioned at 61.87 Mb (solcap_snp_c2_37119, DMv4.03 (Sharma et al. 2013)) and that by Śliwka et al. (2008) is also located at the very bottom end of the same chromosome. The chromosome 3 eye depth QTL–MTA (chr03_60298153) observed here is in strong agreement to the QTL location mentioned in the above two studies. Hara-Skrzypiec et al. (2018) did not detect the major chromosome 10 QTL, however, the most important QTL for eye depth detected in their study was on chromosome 4 (0–33.6 cM; 0–25.6 Mb; see above for cM-to-Mb conversion). The current study reports three QTL-MTAs on chromosome 4 (Table 5) and two (chr04_5763134 and chr04_10456693) of these support the chromosome 4 QTL reported by Hara-Skrzypiec et al. (2018). Zhao et al. (2023) found a QTL for eye depth on chromosome 6 (Chr06A2:25.9–26.4 Mb (Wang et al. 2022)) but the same chromosome QTLs (chr06_40734919 and chr06_40735030, Table 5) detected here were ~ 14 Mb distance apart. Hara-Skrzypiec et al. (2018) identified another eye depth QTL towards the bottom part of chromosome 11 (44.8 cM; 34.2 Mb, DMv6.01; see above for cM-to-Mb conversion), however, all four QTL-MTAs detected in the current study were on the top part of the same chromosome (Table 5). Similarly, the chromosome 12 QTL observed by Lindqvist-Kreuze et al. (2015) in a diploid family of Andean potatoes ranged from 29.08 cM to 44.55 cM (QTL peak at 33.69 cM) while the QTL–MTA detected in the current study is located at the top end of chromosome 12 (chr12_1449252, Table 5). To the best of our knowledge, the chromosomes 7 and 9 QTL-MTAs are novel and not reported previously.

#### Total tubers per plant and tubers per stem

Tuber number is an economically important trait that affects marketable yield and suitability for specific markets. The trait is influenced by many agronomic and environmental factors but tuber number is somewhat genotype dependent (Celis-Gamboa et al. 2003). Previous genetic mapping studies on tuber number have identified QTLs on chromosomes 2, 4, 5, 7, 8 and 10 using biparental (Manrique-Carpintero et al. 2015, 2018; Rak et al. 2017) and association mapping (Schönhals et al. 2016; Zhang et al. 2022) approaches. The QTL-MTAs for tuber number detected in this study map to chromosomes 1, 4 and 9 (chr01_66016265, chr04_63755427 and chr09_50878917; Table 5; Figs. 9, 10; Supplementary Fig. S6). Out of the four QTLs on chromosome 4 reported by Manrique-Carpintero et al. (2018), one is located at the bottom end of the chromosome (143 cM equalling ~ 81.6 Mb; total genetic map length 1299.1 cM; cM-to-Mb conversion procedure as described above) but not so close to the QTL region observed here. Another study (Rak et al. 2017) reporting QTL on chromosome 4 for this trait finds the QTL peak at 81 cM (equalling ~ 57.7 Mb; genetic map length 1041.4 cM; cM-to-Mb conversion procedure as described above) with the closest sequence-based marker (solcap_snp_c2_39439) positioned at 63.55 Mb (DMv4.03 (Sharma et al. 2013)) matching very closely with the chromosome 4 QTL–MTA detected here. Among all other QTLs, the overlapping of chromosome 4 QTL across multiple studies suggests the importance of this locus in regulating tuber number. However, Rak et al. (2017) only observed the chromosome 4 QTL in their biparental mapping analysis and not GWAS conducted as part of the same study citing lack of power for the latter analysis. The current study overcomes this barrier providing a sequence-based QTL–MTA while extending the robustness and applicability of this potentially important chromosome 4 QTL locus in a wider germplasm. In addition to tubers per plant, the current study also investigated GWAS for ***tubers per stem***. Three significant QTL-MTAs on chromosomes 5 and 6 were observed (Table 5; Figs. 9, 10; Supplementary Fig. S6). No previous study reports the genetic analysis for this trait. The QTL for ‘tubers per stem’ and chromosome 1 and 9 QTL for ‘tubers per plant’ traits reported here are novel to the current investigation and provide additional breeding targets for manipulating potato tuber number and yield.

#### Average tuber weight

Tuber weight is one of the main constituents of tuber yield, and together with total tuber number governs the overall productivity of the potato crop. Previous studies report genetic architecture for this trait spanning QTLs on various chromosomes viz., 1, 2, 4, 5, 6, 7 and 10 (Braun et al. 2017; Hara-Skrzypiec et al. 2018; Manrique-Carpintero et al. 2018). GWAS here revealed significant QTL-MTAs for average tuber weight on chromosomes 1 and 2 (Table 5; Figs. 9, 10; Supplementary Fig. S6). Braun et al. (2017) identified QTLs for average tuber weight on chromosome 1 spanning 71.29–77.19 Mb genomic interval (DMv4.03 (Sharma et al. 2013)). Hara-Skrzypiec et al. (2018) identified QTLs for this trait on chromosomes 1, 4, 5 and 6. The genomic interval for their chromosome 1 QTL ranged from ~ 27.6 to 70 Mb (36.2–91.8 cM; total genetic map length 972; cM-to-Mb conversion procedure as described above). The chromosome 1 QTL–MTA (chr01_66991241, Table 5) revealed here lies in the genomic interval reported by this previous study, and is in gross agreement with the genetic locus identified by Braun et al. (2017). Another study (Manrique-Carpintero et al. 2018) reports QTLs on chromosomes 2, 4, 7 and 10 for this trait and the location of chromosome 2 QTLs (~ 53.1 Mb and ~ 59.9 Mb; 93 cM and 105 cM; total genetic map length 1299.1 cM; cM-to-Mb conversion procedure as described above) described by these authors broadly agree to the chromosome 2 QTL–MTA (chr02_40212654, Table 5) detected here.

#### Tuber yield

Tuber yield is the most important target trait for potato crop improvement and remains a prime focus for potato research and breeding. Tuber yield has a complex genetic architecture posing great challenges for breeding higher yielding potato varieties. During the last three decades of genetic mapping using various types of DNA-based markers applied over a number of diploid and tetraploid potato populations, exploiting linkage and association mapping approaches, several QTLs for tuber yield have been reported on chromosomes 1, 2, 4, 5, 6, 7 and 12 (Bonierbale et al. 1993; Bradshaw et al. 2008; Chen et al. 2021; Freyre and Douches 1994; Kreike et al. 1996; Manrique-Carpintero et al. 2015; Marand et al. 2019; McCord et al. 2011; Pereira et al. 2021b; Rosyara et al. 2016; Schafer-Pregl et al. 1998). Schonhals et al. (2017), in their analyses using 90 tetraploid genotypes involving contrasting phenotypes for tuber yield, tuber starch content and tuber starch yield, genotyped using RAD (Restriction-site Associated DNA) sequencing and Infinium 8k Potato SNP Array (Felcher et al. 2012; Hamilton et al. 2011), predicted that at least 200 QTL regions with one or more underlying genes on all chromosomes control the natural variation for these traits. Tuber yield GWAS conducted in the current investigation revealed QTL-MTAs distributed over chromosomes 1, 2, 3, 4, 6, 8 and 12 (Table 5; Figs. 9, 10; Supplementary Fig. S6) overlapping several of the QTL chromosomes mentioned above.

#### Height breadth ratio (HB ratio)

Plant height and breadth are important agronomic traits, affecting architecture and above ground biomass (foliage), the modification of which can improve general plant vigour, yield, ability of stress adaptation and several crop management aspects. The current study performed the genetic analysis on potato canopy architecture (using HB ratio as a proxy) assessed in a tetraploid diversity panel. Significant QTL regions were identified on chromosomes 3, 5, 10 and 12 (Table 5; Figs. 9, 10; Supplementary Fig. S6). The chromosome 5 QTL–MTA identified here (chr05_4944682) is in close vicinity to *St-CDF1* gene (cycling DOF factor, Soltu.DM.05G005140, chr05:4485531–4488495, DMv6.1) known to control plant maturity in potato (Kloosterman et al. 2013). According to Bradshaw et al. (2004) a single locus, at the top end of chromosome 5, with allelic variation for a physiological trait is assumed to affect potato plant height and maturity and indirectly control resistance/susceptibility to blight. The closeness of chromosome 5 QTL to the well described maturity locus corroborates the above assumption associating plant height with maturity, and the additional QTL detected here potentially correspond to further loci regulating these traits.

#### Tuber dry matter

Tuber dry matter, specific gravity, and starch content are highly correlated traits (Houghland 1966; LeClerg 1947), and co-localization of QTLs for these traits is expected (McCord et al. 2011). Considering this the interpretation of QTL results from the studies on these three traits is used interchangeably. Tuber dry matter content, estimated by specific gravity primarily reflecting starch content, is an important agronomic trait for industrial and food processing impacting quality and consistency of the final processed products such as potato chips and French fries. The trait is influenced by genetic and environmental factors including genotype-by-environment interactions (Ruttencutter et al. 1979). Various studies (Bradshaw et al. 2008; Freyre and Douches 1994; Freyre et al. 1994; Li et al. 2019, 2008; Manrique-Carpintero et al. 2015; McCord et al. 2011; Park et al. 2021; Pereira et al. 2021b; Schafer-Pregl et al. 1998; Schönhals et al. 2016) on specific gravity conducted using diploid and tetraploid populations reveal the presence of QTLs for this trait on all 12 potato chromosomes. GWAS for tuber dry matter here detected two QTL-MTAs (chr10_58099346 and chr12_57450585; Table 5; Figs. 9, 10; Supplementary Fig. S6) on chromosomes 10 and 12 which find correspondence with previous studies (Li et al. 2008; Schafer-Pregl et al. 1998). The lack of sequence-based markers and limited resolution of the QTL positions in these two studies prevent a direct comparison with the associations detected in this investigation; however, the position of the chromosome 10 and 12 QTLs reported by these authors (for Li et al. (2008) chromosome 10 QTL only) finds good correspondence with the QTL-MTAs revealed here. Schönhals et al. (2016) also report chromosome 10 MTAs for tuber starch content in the genomic region spanning 50.94–55.85 Mb (DMv4.03 (Sharma et al. 2013)) which is in close vicinity to the chromosome 10 QTL–MTA (chr10_58099346) detected in the current study; however, the chromosome 12 MTAs reported in the two studies are at the opposite ends of the chromosome.

#### QTL hotspots

GWAS performed here identified QTL ‘hotspots’ on all 12 chromosomes with MTAs for multiple traits appearing in close proximity, i.e. within ~ 2 Mb of adjacent MTAs (Table 5; Figs. 9, 10). For example, MTAs for ‘ACB and BLS’ were located together on chromosomes 1, 5, 7, 8 and 10, and for ‘ACB and FSH’ on chromosomes 2, 3, 7 and 10. These traits also shared QTL regions with several other traits such as on chromosome 2 (ACB, BLS, FSH, BRT and SPR; BLS, FSH, ATW and BRT), chromosome 3 (BLS and EYE), chromosome 4 (BLS, SPR and TTU), chromosome 5 (ACB, BLS and SPR), chromosome 6 (ACB, YLD and TPS), chromosome 7 (ACB, BLS, FSH, SPR and EYE), chromosome 8 (FSH and BLK), chromosome 9 (ACB and SPR), chromosome 10 (ACB, FSH and DRM; BLS and HBR), chromosome 11 (FSH and SPR) and chromosome 12 (ACB and EYE, ACB and CSC). Other combinations of QTLs appearing together were on chromosome 1 (SPR, CSC, TTU and ATW), chromosome 3 (SPR and YLD), chromosome 6 (BRT and EYE), chromosome 10 (UNI, EYE and SHP), chromosome 11 (SPR and EYE), and chromosome 12 (SPR and DRM). There were thirty-three cases of identical MTAs shared by more than one trait viz., one among ‘EYE, SHP and UNI’, four between ‘ACB and FSH’, and twenty-eight between ‘SHP and EYE’ (Supplementary Table S9c). All four identical MTAs for ACB and FSH were located on four separate QTL regions (chr2: ~ 32 Mb, chr3: ~ 48 Mb, chr7: 53 Mb and chr10: ~ 1 Mb) while all identical MTAs for SHP and EYE (and one MTA together with UNI) originated from one QTL region only (chr10: ~ 50 Mb).

FSH and ACB show a significant correlation (− 0.26, Fig. 8) and the association of their QTL-MTAs on four distinct chromosomes is intriguing. Similarly, the co-existence of QTL-MTAs for ATW and TTU on chromosome 1 in the region where two QTL-MTAs (at 48.6 Mb and 78.7 Mb) for YLD are also detected (Table 5). ATW displays significant correlations (Fig. 8) with TTU (− 0.68) and YLD (+ 0.63) and this QTL hotspot region on chromosome 1 potentially indicates an important target region for manipulating tuber yield. As we describe in the *Phenotyping* section, the strong correlation (− 0.42) between ACB (scoring, 1 severe) and DRM is interesting and the co-localization of their QTL-MTAs on chromosome 10 further indicates the interaction of genetic factors controlling these two important traits. DRM also shows significant correlations (Fig. 8) with SHP (− 0.24; scoring 1–6, 1 round) and EYE (− 0.27; scoring 1 deep, 9 shallow), and interestingly the significant QTL-MTAs for these two traits lie within 8 Mb distance of DRM QTL–MTA on chromosome 10. SHP and EYE showed a strong correlation (0.47) as stated above and the two traits also displayed co-localization of QTL-MTAs on chromosome 10. This has been well reported in previous studies (Lindqvist-Kreuze et al. 2015; Rosyara et al. 2016) and was further validated here. For one of the MTAs on chromosome 10, these two traits (SHP and EYE) also shared QTL region with UNI which displays a strong correlation with SHP (− 0.42) and the co-localization of MTAs for these three traits is not reported previously to the best of our knowledge. Overall, the QTL hotspots reported here add interesting insights governing these important traits and provide novel targets for potato breeding and research.

## Conclusion

The current study reports the application of genotyping-by-sequencing, a flexible and ‘open’ genotyping approach, in the genetic analysis of cultivated potato using a large and diverse association panel. Moreover, results from use of GBS are compared with the outputs from using a ‘fixed’ SNP genotyping platform. The GBS libraries, after the evaluation of a number of restriction enzyme combinations, were constructed using *Pst*I/*Mse*I restriction enzyme pair yielding SNPs largely confined (~ 90%) to genic or gene-associated region of the genome validating the effectiveness of selecting a methylation-sensitive restriction enzyme (*Pst*I) for constructing GBS libraries.

Genetic analyses conducted using robust sets of GBS and Infinium SNPs did not detect clear separation of genotypes into distinct subpopulations as also reported previously for cultivated potato. Nevertheless, the analysis using both SNP sets indicated the presence of three subpopulations within the association panel and, between the two approaches, GBS SNPs were able to discriminate the three subgroups more effectively. The LD decay rates, assessed at the whole chromosome level, obtained here are comparable to those reported previously in potato, however, the estimates using GBS SNPs were significantly sharper than those displayed by the Infinium SNPs. The difference was more pronounced for chromosome 8 for which some of the previous studies as well as the current Infinium-based analyses revealed extremely conserved levels of LD decay as compared to other chromosomes, however, the chromosome 8 LD decay estimate using GBS SNPs was in good agreement with the levels observed for other chromosomes.

The germplasm panel displayed a broad range of phenotypic diversity for most traits analysed here with a wide heritability range and significant correlations between several trait pairs. GWAS were performed using a combined (GBS and Infinium) set of 30,363 SNPs yielding 1181 significant MTAs covering all traits included in the study. These were further converted to 189 unique QTL-MTAs following LD-based filtering and retaining MTAs with highest significant *p* value per LD window interval; and also keeping only the most significant MTA for SNPs appearing with multiple gene action models. The majority of QTL-MTAs obtained were from GBS SNPs highlighting their proximity to the causal variation and increased marker saturation and resolution achieved through GBS. This potentially also illustrates the utility of marker-dense de novo genotyping platforms in overcoming ascertainment bias and providing a more accurate correction for different levels of relatedness in GWAS models.

The study provides robust and LD-based filtered set of most significant QTL-MTAs for sixteen important production, agronomic and processing traits in potato. It is worth noting that most previous studies for these traits, besides a lack of any previous study for some of the traits, were performed on diploid biparental populations. The current study, however, deployed a large tetraploid varietal panel representing a larger diversity of the cultivated potato gene pool suggesting that the loci discovered here, especially those in common between the previous studies, may be the main ones controlling these highly important traits especially in the context of European potato germplasm. Moreover, the previous studies largely used low-throughput non-sequenced-tagged markers with a few employing fixed SNP platforms, whereas the current study exploited genome-wide genotyping and de novo SNP discovery simultaneously; and the reported sequence-tagged MTAs are likely to have higher transferability across a wider germplasm and increased utility for expediting genomics-assisted breeding and developing diagnostic markers for the studied traits.

GWAS here detected QTL hotspots for several traits spanning all 12 chromosomes covering known, such as for tuber shape and eye depth, as well as novel regions. Some of these traits also show significant correlations, and further efforts will be needed to unravel whether these trait QTL hotspots are governed by pleiotropic effects or independently acting but closely linked loci, or a combination of both. The observations further highlight the challenges in identifying functional genes underlying these QTL if the trait is studied in isolation of the other affected traits, notwithstanding the interplay of multiple mechanisms involved in trait inheritance. Overall, the genetic architecture for the studied traits and the QTL hotspots reported here illustrate the challenges faced in understanding and breeding for complex traits, but also provide a sound foundation for overcoming the hurdles encountered in improving such traits.

GWAS can be performed for a greater number of traits in a single study as opposed to developing bespoke populations for every single trait in traditional biparental mapping. The latter requires more resources, is time consuming and only displays diversity present in the two parents rather than a larger allele repertoire scanned through GWAS. Sequence-based genotyping performed here, exploiting methylation-sensitive-GBS, targeted largely the euchromatic part of the genome with the increased ability, in a cost-effective manner, to saturate any population of interest with thousands of markers. The main finding reveals the effectiveness of flexible sequence-based genotyping over fixed SNP platform for identifying significant marker–trait associations with higher precision and resolution as well as detection of additional QTL.

### Supplementary Information

Below is the link to the electronic supplementary material.

Supplementary Fig. S1 Screeplot from the Principal Component Analysis (PCA) displaying the number of principal components versus their corresponding eigenvalues. PCA performed using (a) GBS SNPs and (b) Infinium array SNPs (PNG 1004 KB)

Supplementary Fig. S2 Bayesian information criterion (BIC) statistical measure of goodness of fit curve for detecting optimal number of clusters (k-means) or subpopulations (Q) in the GWAS panel obtained using (a) GBS SNPs and (b) Infinium array SNPs (PNG 851 KB)

Supplementary Fig. S3 Illustration of relationships (level of discrimination) among GWAS panel subpopulation groups (Q) using DAPC (Discriminant Analysis of Principal Components) single-axis density plot. DAPC performed using (a) GBS SNPs and (b) Infinium array SNPs (TIF 24249 KB)

Supplementary Fig. S4 The distribution of individual trait values and residuals (PNG 2686 KB)

Supplementary Fig. S5 Q-Q plots comparing the inflation of p-values for the four principal GWAS models deployed for each genetic (gene action) model for all 16 traits. Red circles: Naïve model; Green squares: K model; Blue diamonds: Q model; and Black triangles: QK model. Red line indicates p-values under the expected normal distribution (PNG 7777 KB)

Supplementary Fig. S6 Trait-wise combined Manhattan plots from all genetic (gene action) models performed for each GWAS statistical model. GWAS significance thresholds (dashed lines), specific to each trait, are derived using Bonferroni-type multiple testing correction method “M.eff” (with genome-wide α = 0.05). The GWAS significance threshold varies across different genetic (gene action) models, therefore, only the most stringent value obtained among these models is plotted in the combined Manhattan plots (PNG 2710 KB)

Supplementary Table S1 List of all tetraploid potato genotypes included in the study (XLSX 15 KB)

Supplementary Table S2 Details of traits phenotyped in each environment (XLSX 12 KB)

Supplementary Table S3 List of barcodes employed to construct 24-plex GBS libraries (XLSX 10 KB)

Supplementary Table S4 List of genotypes included in construction of GBS libraries (XLSX 22 KB)

Supplementary Table S5 Raw read data processing summary for 16 GBS libraries (XLSX 16 KB)

Supplementary Table S6 List of genes overlapping GBS SNPs including impact category count (XLSX 1814 KB)

Supplementary Table S7 GWAS panel population group (cluster) membership details (XLSX 25 KB)

Supplementary Table S8 Genomic control inflation factor (λ_GC_) values for each trait and 'GWAS genetic model x statistical model' combination (XLSX 23 KB)

Supplementary Table S9 List of significant marker–trait associations (MTAs) from different result categories: (a) List of significant MTAs from K and QK models. MTAs appearing for all gene action models are included; (b) List of most significant MTAs per QTL (QTL-MTAs) from K and QK models. QTL-MTAs appearing for all gene action models are included; (c) List of thirty-three 'most significant MTAs' found to be identical between multiple traits. Each alternate colour-shaded category represents a unique MTA (XLSX 114 KB)

## Data Availability

The genotype and phenotype data files are available at figshare: 10.6084/m9.figshare.25601394. Sequence data have been deposited in the European Nucleotide Archive (ENA) at EMBL-EBI under accession number PRJEB74779 (https://www.ebi.ac.uk/ena/browser/view/xyz).
