# Supplementary figures and images for: Genotyping-by-sequencing targets genic regions and improves resolution of genome-wide association studies in autotetraploid potato
Source: Theor Appl Genet. 2024 Jul 9;137(8):180. doi: 10.1007/s00122-024-04651-8 (PMC11233353; doi:10.1007/s00122-024-04651-8)

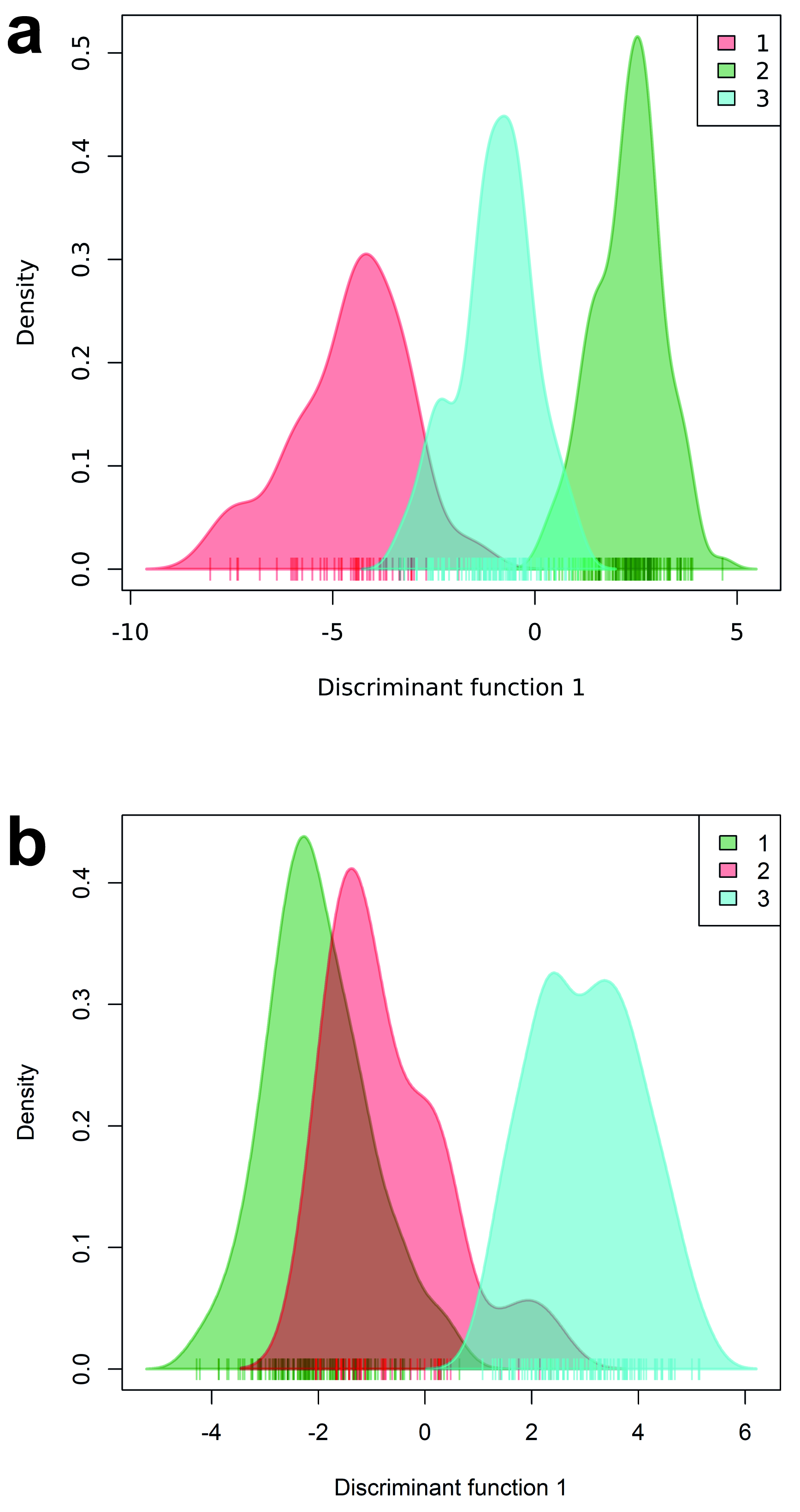

Supplement: Supplementary file 3 — Supplementary Fig. S3 Illustration of relationships (level of discrimination) among GWAS panel subpopulation groups (Q) using DAPC (Discriminant Analysis of Principal Components) single-axis density plot. DAPC performed using (a) GBS SNPs and (b) Infinium array SNPs (TIF 24249 KB) [file 122_2024_4651_MOESM3_ESM.tif]

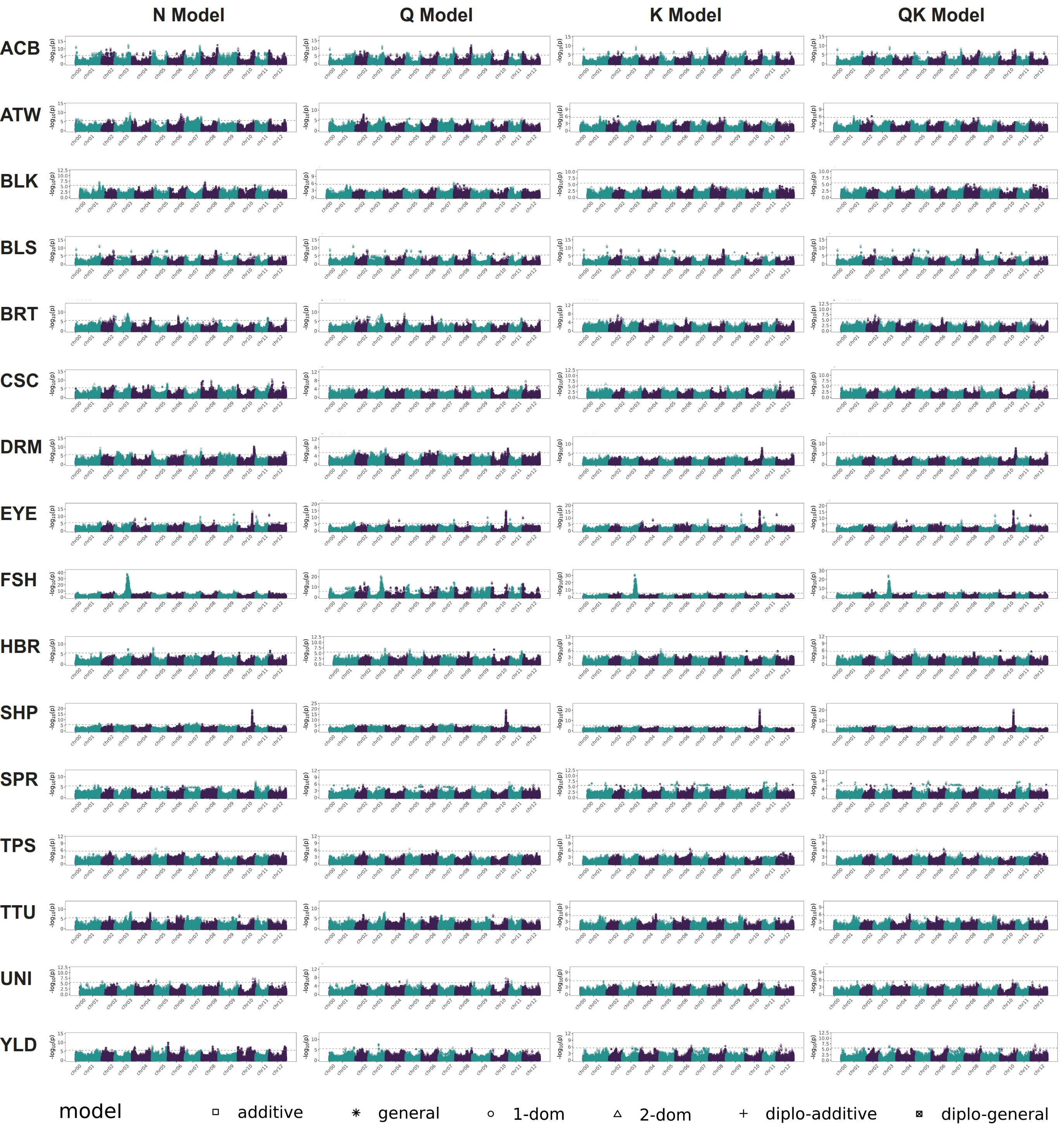

Supplement: Supplementary file 6 — Supplementary Fig. S6 Trait-wise combined Manhattan plots from all genetic (gene action) models performed for each GWAS statistical model. GWAS significance thresholds (dashed lines), specific to each trait, are derived using Bonferroni-type multiple testing correction method “M.eff” (with genome-wide α = 0.05). The GWAS significance threshold varies across different genetic (gene action) models, therefore, only the most stringent value obtained among these models is plotted in the combined Manhattan plots (PNG 2710 KB) [file 122_2024_4651_MOESM6_ESM.png]
